# Characterization of Liquid Dosage Forms of Atenolol and Enalapril Maleate for Oral and Enteral Feeding Administration

**DOI:** 10.3390/ph17081052

**Published:** 2024-08-09

**Authors:** Sandra Mota, Ana Torres, Clara Quintas, António M. Peres, Nuno Ferreiro, Rebeca Cruz, Helena Ferreira, Isabel F. Almeida, Susana Casal

**Affiliations:** 1 Associate Laboratory i4HB, Institute for Health and Bioeconomy, Faculty of Pharmacy, University of Porto, 4050-313 Porto, Portugal; up201608486@up.pt (S.M.); up201706122@up.pt (A.T.); claraquintas@ff.up.pt (C.Q.); 2 UCIBIO—Applied Molecular Biosciences Unit, Laboratory of Technology, Department of Drug Sciences, Faculty of Pharmacy, University of Porto, 4050-313 Porto, Portugal; 3 UCIBIO—Applied Molecular Biosciences Unit, Laboratory of Pharmacology, Department of Drug Sciences, Faculty of Pharmacy, University of Porto, 4050-313 Porto, Portugal; 4 CIMO, LA SusTEC, Instituto Politécnico de Bragança, 5300-253 Bragança, Portugal; peres@ipb.pt (A.M.P.); nuno.ferreiro@ipb.pt (N.F.); 5 Associated Laboratory for Green Chemistry (LAQV) of the Network of Chemistry and Technology (REQUIMTE), Laboratory of Bromatology and Hydrology, Department of Chemical Sciences, Faculty of Pharmacy, University of Porto, 4050-313 Porto, Portugal; rcruz@ff.up.pt (R.C.); sucasal@ff.up.pt (S.C.); 6 UCIBIO–Applied Molecular Biosciences, Laboratory of Microbiology, Department of Biological Sciences, Faculty of Pharmacy, University of Porto, 4050-313 Porto, Portugal

**Keywords:** stability, compounding, oral formulations, atenolol, enalapril maleate, enteral feeding, liquid dosage forms

## Abstract

The limited availability of pharmaceutical formulations tailored for cardiovascular diseases in both pediatric and geriatric populations generates the need for compounded dosage forms to guarantee precise dosing and medication adherence. This study aimed to analyze the physicochemical properties and stability of formulations of atenolol and enalapril maleate prepared with a proprietary oral vehicle, SuspendIt^®^. To this end, palatability, injectability, pH, rheological behavior, and physical, microbiological, and chemical stability over a 180-day storage period at 25 °C and 5 °C were evaluated. Injectability tests confirmed the suitable use of both formulations for administration through enteral feeding tubes. By using a potentiometric electronic tongue, it was confirmed that the SuspendIt^®^ vehicle effectively served as a bitter-blocking strategy for atenolol and enalapril maleate. Adequate stability throughout the storage period was confirmed in terms of the mechanical properties, pH, and effectiveness of the preservative system. The atenolol concentration remained above 90% of the initial amount, while the concentration of enalapril maleate decreased to 88% after 90 days of storage at 25 °C. In summary, the atenolol formulation maintained suitable chemical, physical, and microbiological stability after 180 days at both storage temperatures, while the enalapril maleate formulation remained stable up to 60 days at 25 °C and for 180 days at 5 °C.

## 1. Introduction

Compounding medicines are important therapeutic options in all areas of medicine and are essential to the provision of healthcare [1]. Their use can overcome pitfalls related to limited dosage forms, limited dosages and strengths, limited orphan medicines, the need for alternative raw materials, and the need for specific combinations of drugs. In addition, they may serve as an alternative in cases of drug shortages or when pharmaceutical industries discontinue certain drugs due to waning interest. Extemporaneous preparations of targeted cardiovascular medications, such as enalapril maleate or atenolol, are typically necessary for particular demographic groups or niches, such as the elderly and children. 

Enalapril is an ethyl ester prodrug converted to its active metabolite enalaprilat, which inhibits angiotensin-converting enzyme (ACE), preventing the formation of angiotensin II and inducing vasodilation. It reduces arterial pressure, afterload, blood volume, and preload, thereby inhibiting cardiac and vascular remodeling. Enalapril is used to treat hypertension, chronic heart failure [2], and chronic kidney disease with proteinuria [3]. Although not fully understood, atenolol appears to exert its therapeutic effect by preventing activation of β1 adrenoceptors in the heart, leading to negative inotropic and chronotropic effects, reducing cardiac output and arterial pressure. The reduction in cardiac workload makes it effective in ischemic coronary disease, while its negative chronotropic and dromotropic effects are the rational basis for the treatment of arrhythmias [4,5,6]. 

Both enalapril maleate and atenolol are commercially available in tablets at fixed dosages (enalapril, 2.5–20 mg; atenolol, 25–100 mg), which poses some challenges to ensuring accurate dose and medication adherence in specific populations, such as pediatrics or the elderly. The pediatric population is heterogeneous, ranging from preterm newborns to adolescents, necessitating dose adjustments based on their weight and age [2,7,8,9]. Additionally, regardless of the patient’s age, the presence of renal insufficiency also demands dose reductions for both enalapril maleate and atenolol [7,8,10]. Therefore, providing alternative options to oral solid dosage forms, such as the development of liquid dosage forms to deliver these drugs, is necessary to ensure accurate dose adjustments based on a patient’s weight, age, and renal function, as well as flexibility as the child grows, thereby reducing the risk of underdosing or overdosing. Furthermore, many children have limited or inconsistent swallowing abilities, as do many elderly individuals, due to age- or disease-related dysphagia or odynophagia [11]. Liquid pharmaceutical forms for oral administration may enhance medication adherence due to the facility of administration, which reduces the risk of choking [12]. In addition to oral administration, patients with neurological conditions that affect swallowing, severe dysphagia, or who are critically ill, such as those who are comatose, may require the administration of medications via enteral feeding tubes [13,14]. Flexibility in dose adjustment is crucial for these patients, who may need smaller doses. Liquid forms also facilitate drug absorption in these patients, often with compromised digestive processes, and reduce the risk of clogging the feeding tube, ensuring that they can receive their necessary medications safely and effectively, decreasing the complications associated with the cross-contamination from sharing of tablet crushing devices and the risks of occupational exposure to drug powders through inappropriate handling [15]. Despite the benefits, liquid oral formulations can also present challenges that must be addressed. These can include stability issues due to their aqueous nature, bioavailability concerns, palatability, and the convenience of transport and administration [16,17]. Overcoming these challenges requires the meticulous selection of excipients and innovative formulation strategies.

In liquid dosage form preparations, the vehicle consists of one or more excipients that carry the active substances [18]. Several oral vehicles are available, differing in their physicochemical and organoleptic properties [19,20]. The selection of an optimal oral vehicle is critical to safeguarding the stability, palatability, and therapeutic effectiveness of drugs [21,22]. Additionally, ongoing research and development of new oral vehicles are needed to address the specific requirements of different patient populations. For instance, there is currently a lack of commercially available liquid drug products specifically formulated for tube-feeding purposes, and compounding medicines often present short beyond-use dates [23,24]. As mentioned above, both enalapril and atenolol are often prescribed for chronic conditions and therefore require prolonged treatment. Therefore, the stability of extemporaneous formulations becomes a critical factor, and the development of products with extended shelf life is an advantage. Furthermore, it is important to ensure that these active substances are correctly solubilized in liquid formulations and remain so under physiological conditions. Atenolol, with a pKa value of 9.6, is sparingly to slightly soluble in water [25]. Its solubility increases in acidic media at pH 1.2 compared to slightly basic media at pH 7.4 [26]. Enalapril maleate is also sparingly soluble in water, exhibiting greater solubility in more basic media (pH 7.2) compared to acidic media (pH 4) [25,27].

SuspendIt^®^ is an aqueous oral vehicle compatible with all feeding tubes that employs a synergistic polymer complex exhibiting a thixotropic behavior [28]. This characteristic facilitates the redispersion of drugs upon agitation, thereby minimizing sedimentation [28]. Comprising a natural sweetener derived from monk fruit, SuspendIt^®^ is particularly well-suited for patients needing formulations devoid of sugar, casein, gluten, and parabens and other potentially harmful excipients for the pediatric population [28,29]. Several stability studies of oral formulations using SuspendIt^®^ as a vehicle have already been conducted. Notable examples include oral suspensions combining trimethoprim and sulfadiazine, as well as oral suspensions of spironolactone, metronidazole, ursodiol, amlodipine besylate, and allopurinol [30,31,32,33,34,35]. Additionally, oral solutions containing clindamycin and naltrexone hydrochloride have also been investigated [36,37].

The aim of this study was to determine the chemical, physical, and microbiological stability of atenolol and enalapril maleate formulations prepared with SuspendIt^®^ when stored at 25 °C and 5 °C for an extended period (180 days). Suitability for oral (palatability) and enteral feeding administration (injectability) was also evaluated.

## 2. Results

### 2.1. Preliminary Characterization of the SuspendIt^®^ Vehicle and the Cardiovascular Oral Formulations

#### 2.1.1. pH

The pH values were similar for the vehicle SuspendIt^®^ (5.00 ± 0.03) and enalapril maleate formulation (4.95 ± 0.01), while a slightly lower pH was found for atenolol liquid preparation (4.65 ± 0.00). The cardiovascular formulations were prepared with the mentioned vehicle.

#### 2.1.2. Rheological Properties

##### Flow Behavior

**Figure 1:**
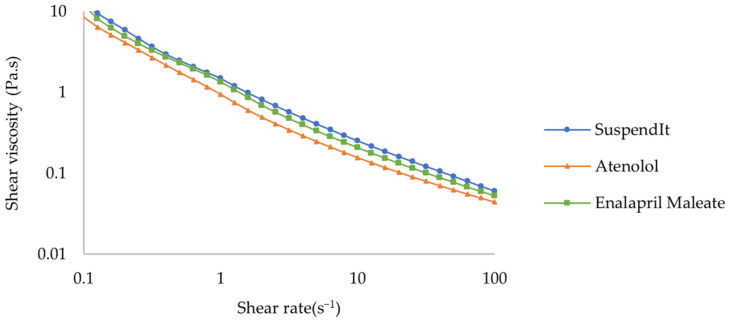
Flow curve of SuspendIt^®^, atenolol, and enalapril maleate formulations.

**Figure 2:**
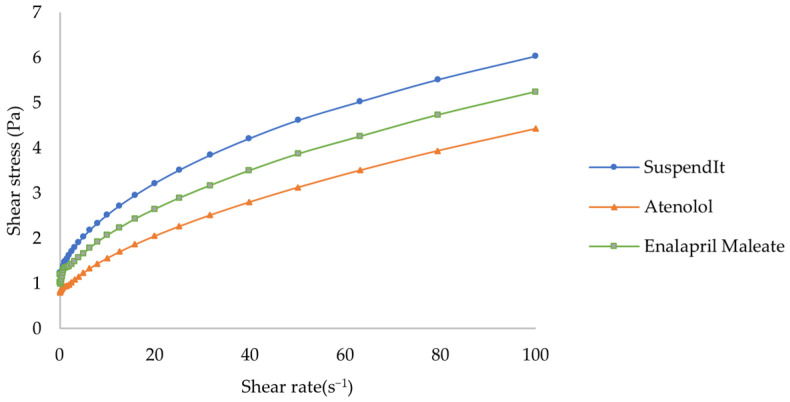
Rheogram of SuspendIt^®^, atenolol, and enalapril maleate formulations.

The apparent viscosity decreased with increased shear rate for SuspendIt^®^ and for both atenolol and enalapril maleate formulations, which is typical of shear-thinning liquids (Figure 1 and Figure 2).

The Hershel-Bulkley model provided a good fit for flow curves. The consistency coefficient was lower for the cardiovascular formulations compared with the vehicle (Table 1). The flow indexes were lower than 1, thus confirming the shear-thinning behavior. Both formulations and the vehicle presented an apparent yield stress. The yield stress represents the stress threshold necessary to induce flow, playing an important role in the processing, physical stability, and practical application of liquid products [38].

##### Thixotropy

**Figure 3:**
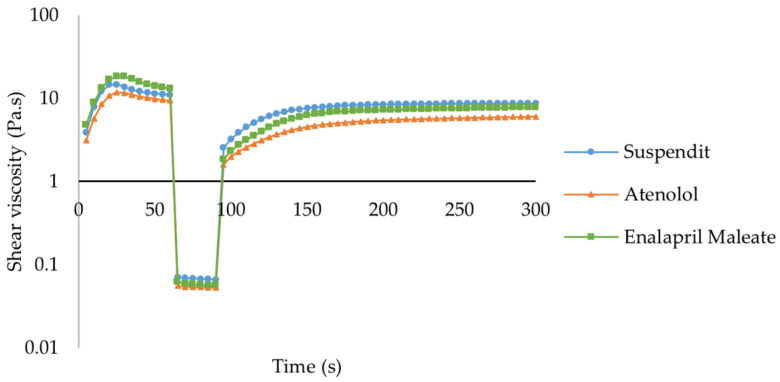
Thixotropic behavior of SuspendIt^®^, atenolol, and enalapril maleate formulations.

SuspendIt^®^ and both atenolol and enalapril maleate formulations are thixotropic fluids since their initial viscosity decreases over time and after applying a shear [39]. After the external force is removed, the initial viscosity is recovered in a time-dependent way [39,40]. Thixotropic behavior is invariably associated with shear-thinning flow behavior [39,40]. The recovery period for both formulations was slightly higher than for SuspendIt^®^ alone, while the recovery rate was slightly lower (Figure 3, Table 2).

##### Amplitude Sweep—Linear Viscoelastic Region (LVER)

**Figure 4:**
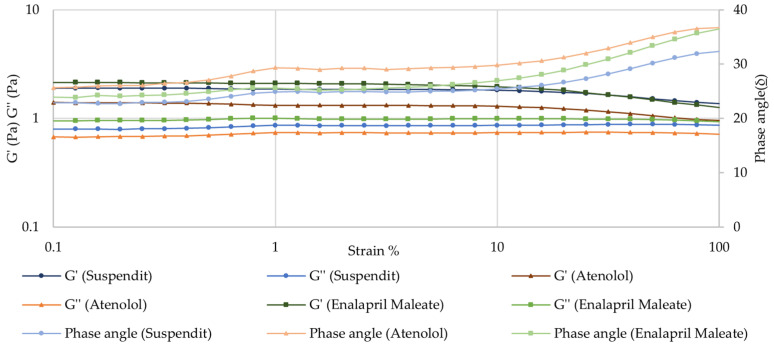
Amplitude sweep for SuspendIt^®^, atenolol, and enalapril maleate formulations.

The amplitude sweep test allows the determination of the linear viscoelastic region (LVER). Beyond a critical strain level, the material’s behavior is non-linear, and the storage modulus (G′) declines. Increasing the strain above the critical strain disrupts the network structure. For both atenolol and enalapril maleate formulations, the LVER was similar to the vehicle—SuspendIt^®^ (Figure 4). From 10% strain, the material becomes progressively more fluid-like, G′ declines, and thus further tests to study the viscoelasticity of these formulations should be performed at lower deformations within LVER. Below 10% strain, the structure is intact, the material behaves solid-like, and G′ > G″, indicating that the material is highly structured. The strength of the colloidal forces is reflected by tan δ = (G″/G′). For the atenolol formulation, the tan δ was about 0.58, while for SuspendIt^®^ and for the enalapril formulation, the tan δ was about 0.47. A tan δ value of less than 1, as observed in the formulations under study, indicates strong particle association driven by colloidal forces [38]. For a system to maintain stability, an intermediate value of tan δ is optimal [38].

##### Frequency Sweep (Mechanical Spectrum)

**Figure 5:**
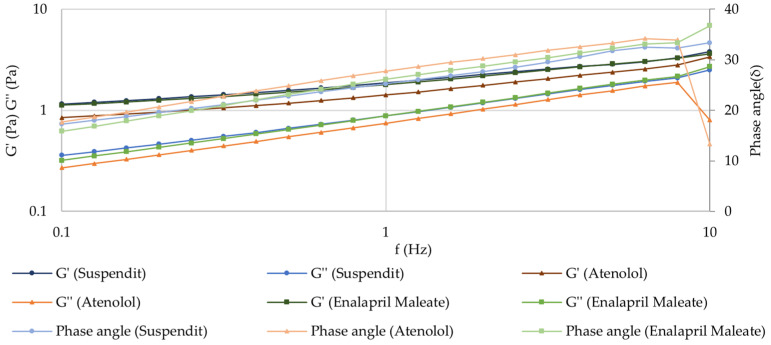
Mechanical spectrum of SuspendIt^®^, atenolol, and enalapril maleate formulations.

The atenolol and enalapril maleate formulations presented a solid-like behavior in the fragment of the spectrum observed, where the storage modulus (G′) was higher than the loss modulus (G″) over the whole frequency range, and the phase angle was lower than 45° [41]. This is the typical behavior of viscoelastic polymeric materials in the plateau zone. No crossover point was observed across the tested frequencies, indicating that the formulations exhibit entangled polymeric networks [41,42]. The observed frequency-dependence of G′ reflects a moderately structured material [41]. The results also demonstrate that the compounding formulation has similar viscoelastic properties as the SuspendIt^®^ vehicle, meaning that their inner structure is similar (Figure 5).

#### 2.1.3. Injectability

**Figure 6:**
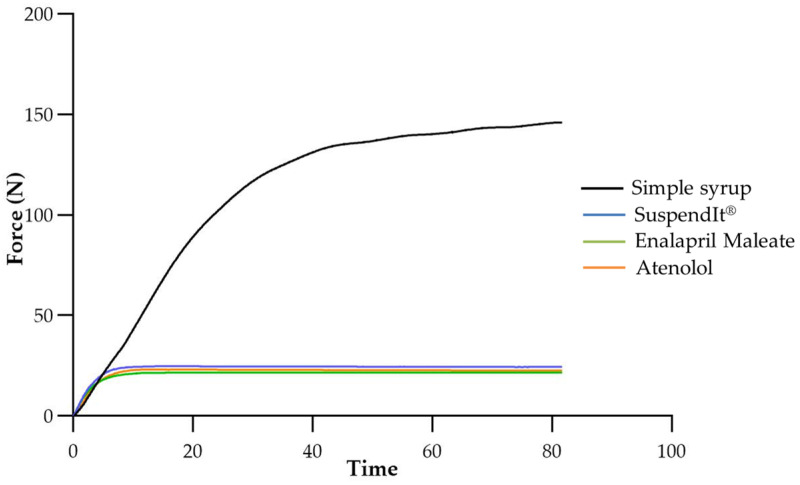
Injectability texturogram obtained for the studied formulations.

The injectability test consisted of measuring the force required to promote the flow of both vehicles and suspensions using a syringe connected to a feeding tube (NeoMed^®^ Enteral Feeding). The selected feeding tube was specifically designed for administration to neonatal and pediatric patients, facilitating nutritional delivery via nasal or oral gastric routes. Simple syrup was chosen as a comparator due to its wide use as a vehicle for dissolving active pharmaceutical ingredients in the preparation of compounded oral medications, which is relevant in hospital settings [43]. The force needed to inject simple syrup was about seven times higher than for the formulations in which the vehicle is SuspendIt^®^. The shear-thinning properties of the SuspendIt^®^ formulations facilitated injection through the feeding tube, in contrast to simple syrup, which is a Newtonian fluid. The force needed to inject SuspendIt^®^ was slightly higher than for the oral formulations of atenolol and enalapril maleate (Figure 6 and Table 3). The variation in injectability among the different SuspendIt^®^ formulations can be attributed to their distinct consistency indexes, with the SuspendIt^®^ vehicle exhibiting a higher consistency index. The tested cardiovascular formulations are thus easy to administer through feeding tubes, even those with a low diameter (outer diameter: 2.2 mm) adapted to neonates and infants. 

#### 2.1.4. Evaluation of the SuspendIt^®^ Oral Suspending Vehicle as a Bitter Bloc for Atenolol and Enalapril Maleate

The effectiveness of SuspendIt^®^ and the atenolol vehicle in masking the bitterness of atenolol and enalapril maleate was assessed qualitatively using a laboratory-developed potentiometric electronic tongue (E-tongue), which was previously built by the research team [17]. The potentiometric signal profiles generated by the 40 lipid sensor membranes of the E-tongue during the analysis of atenolol or enalapril maleate aqueous or vehicle solutions were used to construct supervised multivariate classification models. Signal intensity varied across sensors (40 sensors ranging from S1:1 to S1:20 and S2:1 to S2:20) and solutions under study: 0.1–336 mV for atenolol aqueous solution, 2–337 mV for atenolol in atenolol vehicles, and 0.7–381 mV for atenolol vehicles; 1–410 mV for enalapril maleate aqueous solution, 0.7–388 mV for enalapril maleate, and 0.5–386 mV for SuspendIt^®^ vehicles. The varying signal profiles and intensities may be ascribed to the diverse electrostatic and/or hydrophobic interactions potentially established between the E-tongue sensor membranes, characterized by negative/positive polarities and polar/non-polar regions, and the hydroxyl, carboxyl, and/or amine functional groups present in atenolol and enalapril maleate, as well as in the constituents of the SuspendIt^®^ vehicles.

**Figure 7:**
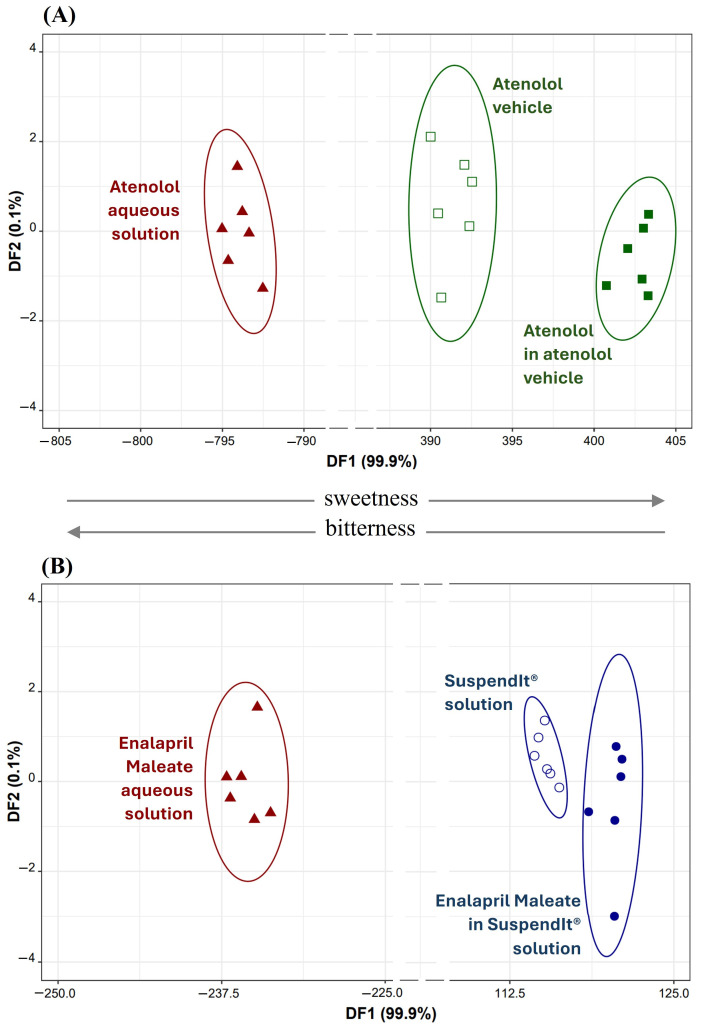
(**A**) Atenolol: 2D LDA plot based on the signals’ profiles of nine sensors of the E-tongue (S1:6, S1:13, S1:17, S1:18, S2:1, S2:5, S2:8, S2:12, and S2:15). (**B**) Enalapril Maleate: 2D LDA plot based on the signals’ profiles of eight sensors of the E-tongue (S1:3, S1:11, S1:13, S1:19, S2:9, S2:11, S2:12, and S2:15).

Different linear discriminant (LDA) models were established based on the E-tongue fingerprints, seeking to assess the bitter block effect of the atenolol vehicle or SuspendIt^®^ vehicle on atenolol or enalapril maleate formulations. This aimed to compare them with the bitter taste of their respective aqueous solutions (used as positive controls) and the pure vehicles (used as negative controls, i.e., sweet solutions). In both cases, LDA models comprising two discriminant functions explained 100% of the total data variability. For atenolol, the model used potentiometric signals from nine sensors (S1:6, S1:13, S1:17, S1:18, S2:1, S2:5, S2:8, S2:12, and S2:15) selected by the simulated annealing (SA) variable selection algorithm. The LDA-SA-E-tongue model exhibited a sensitivity of 100% for both training (Figure 7A) and internal validation (leave-one-out cross-validation, LOO-CV) procedures. For enalapril maleate, the supervised classification model employed signals from eight sensors (S1:3, S1:11, S1:13, S1:19, S2:9, S2:11, S2:12, and S2:15) selected by the SA algorithm. The LDA-SA-E-tongue model correctly classified all train set solutions (100% sensitivity, Figure 7B) and 95% of solutions in the LOO-CV procedure, with only one enalapril maleate solution in the SuspendIt^®^ vehicle being misclassified as if it were a SuspendIt^®^ vehicle without the drug.

From the data depicted in Figure 7, it is evident that both atenolol and enalapril maleate, represented by their aqueous solutions (which are typically bitter), are consistently positioned in the negative region of the respective first discriminant function (DF1). Conversely, solutions containing these drugs in the atenolol vehicle or SuspendIt^®^ vehicle, as well as the pure vehicle solutions (known to be sweeter), are consistently placed in the positive region of DF1. This observation confirms the efficacy of the used vehicles in masking or blocking the bitter taste associated with atenolol or enalapril maleate. Additionally, by analyzing the coordinates of each drug’s group centroid (aqueous versus vehicle solution), it became feasible to compute the distance between them. For atenolol, the distance between the groups was measured at 1197 arbitrary units, whereas for enalapril maleate, it was significantly lower at 356 arbitrary units, indicating a more than threefold difference. This difference suggests that the atenolol vehicle was more successful in suppressing the bitterness of atenolol compared to the SuspendIt^®^ vehicle for enalapril maleate. Furthermore, the placement of the groups (negative versus positive region of DF1) and the positive/negative contribution of each selected sensor included in DF1 enable us to deduce that certain sensors (S1:3, S1:19, S2:1, S2:8, S2:9, and S2:15) are more prone to detecting bitterness, whereas others (S1:6, S1:11, S1:13, S1:17, S1:18, S2:5, and S2:12) demonstrate a higher affinity for detecting sweetness. It is worth noting that sensors S1:11 and S2:11, although having the same composition, were included in the same LDA model, possibly due to differences in their physical properties, such as porosity and transparency, which could arise due to the drop-by-drop technique employed during the device fabrication, as previously discussed [17].

**Figure 8:**
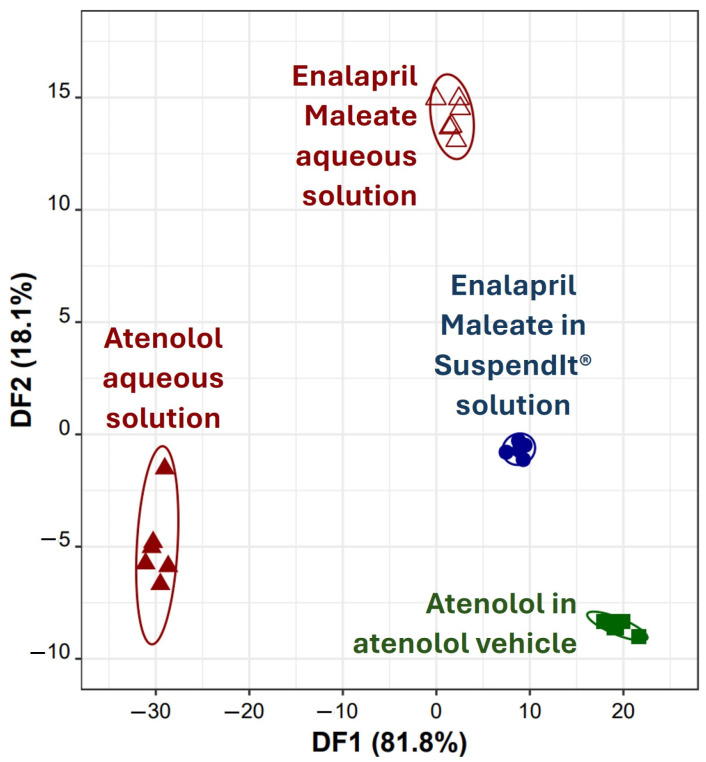
Discrimination of atenolol and enalapril maleate solutions using the E-tongue: 2D LDA plot based on the signals’ profiles of three sensors of the E-tongue (S1:10, S2:3, and S2:5).

Lastly, it was explored the capability of the developed taste sensor device to distinguish between aqueous solutions of two drugs, atenolol and enalapril maleate, as well as their solutions in SuspendIt^®^ vehicles simultaneously. To achieve this, a novel LDA-SA-E-tongue classification model was developed. This model, employing potentiometric signals from three E-tongue sensors (S1:10, S2:3, and S2:5) selected via the SA algorithm, comprised three DFs that explained 81.8%, 18.1%, and 0.1% of the data variability. The model allowed accurate classification of all samples during both training (Figure 8) and LOO-CV procedures. These findings demonstrate the effectiveness of the E-tongue in distinguishing the varying bitterness/sweetness levels among the four oral formulations investigated: atenolol and enalapril maleate in water (likely differing in bitterness intensity) and the oral suspensions of both drugs in SuspendIt^®^ vehicles (with differing sweetness intensities). Analysis of sensor contributions to the DFs suggests that S2:3 (negatively contributing to DF1) primarily detected bitterness, while S1:10 and S2:5 (positively contributing to DF1) exhibited higher sensitivity to sweetness. Particularly, the substantial difference between the centroids of atenolol aqueous solution and atenolol in the atenolol vehicle (distance = 49 arbitrary units) compared to that of enalapril maleate aqueous solution and enalapril maleate in the SuspendIt^®^ vehicle (distance = 16 arbitrary units) suggests that while both vehicles effectively block bitterness, the atenolol vehicle demonstrates superior effectiveness in masking bitterness compared to the SuspendIt^®^ vehicle, as evidenced by the larger centroid distance. In addition to the natural sweetener derived from monk fruit found in SuspendIt^®^, the atenolol vehicle contains two additional sweeteners: acesulfame potassium and steviol glycosides (95%). These added sweeteners likely contribute to its enhanced capability to mask the bitter taste of the active ingredients.

### 2.2. Stability Study of the Oral Formulations

#### 2.2.1. Organoleptic Characteristics

The appearance and odor of the formulations remained unchanged throughout the period of analysis except for a slight yellowing observed at 25 °C after 180 days of storage for both atenolol and enalapril maleate formulations (Figures S6 and S7) compared to their appearance and odor at time zero.

#### 2.2.2. pH

After 180 days of storage, the pH of both formulations remained stable with less than 5% variation (Table 4). Also, no relevant differences were observed between the two storage temperatures tested.

#### 2.2.3. Rheological Properties

##### Flow Behavior

**Figure 9:**
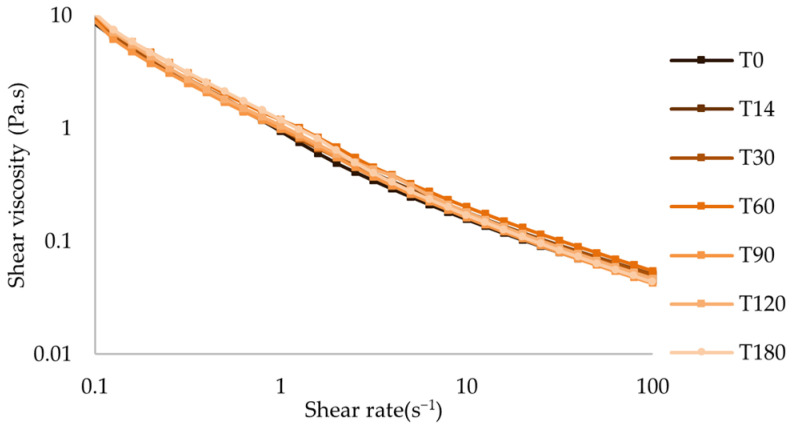
Flow curve of atenolol oral formulation after storage at 25 °C.

The shear-thinning behavior of the oral formulations remained unchanged throughout the period of analysis, with slight variations in apparent viscosity (Figure 9 and Figure S8–S10). After 180 days of storage at both temperatures, the variation in all Hershel-Bulkley parameters was roughly lower than 15% (Table 5, Table 6, Table 7 and Table 8).

##### Thixotropy

**Figure 10:**
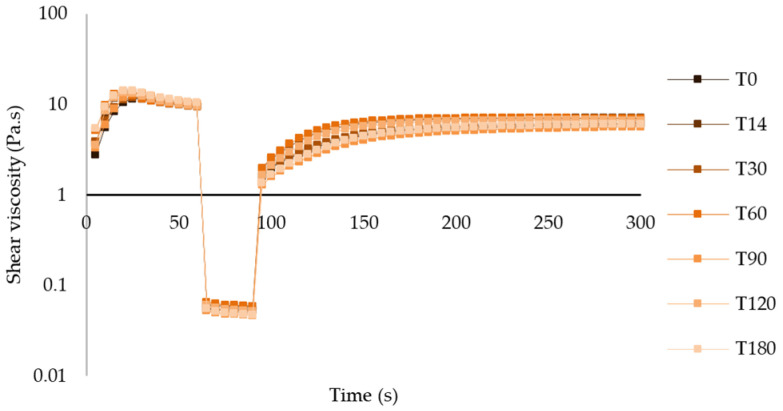
Thixotropic behavior of atenolol oral formulation after storage at 25 °C.

Analyzing the graphs of the thixotropic behavior of both atenolol and enalapril maleate formulations at both temperatures, the recovery behavior after a shear period was similar throughout the period of analysis, with slight variations in the recovery rate and period (Figure 10 and Figures S11–S13, Tables S3–S6).

##### Amplitude Sweep—Linear Viscoelastic Region (LVER)

**Figure 11:**
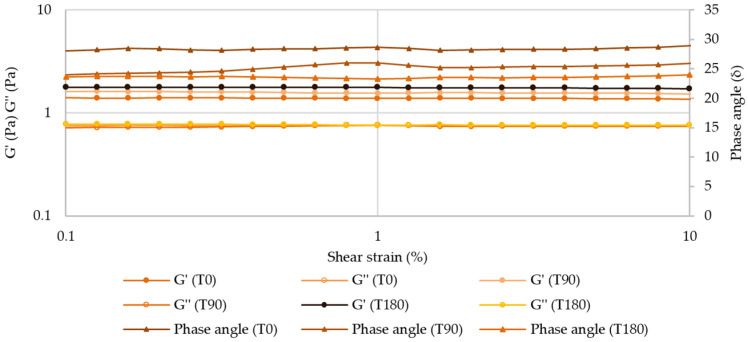
Amplitude sweep for atenolol oral formulation after 180 days of storage at 25 °C.

After 180 days, there were no relevant changes in the LVER of atenolol and enalapril maleate formulations for both temperatures, with critical strains around 10% (Figure 11 and Figures S14–S16).

##### Frequency Sweep (Mechanical Spectrum)

**Figure 12:**
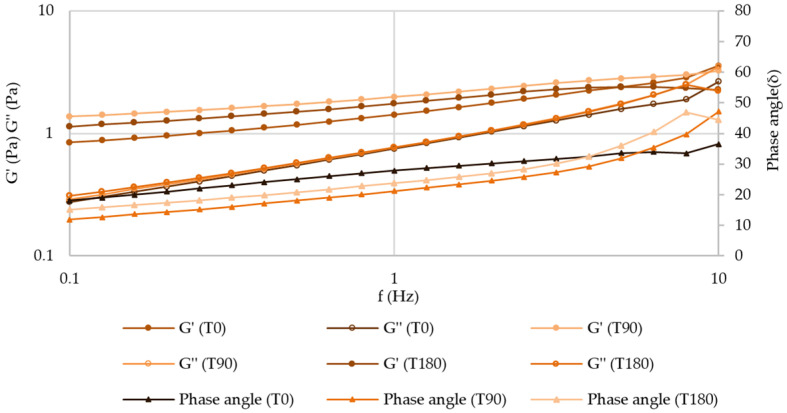
Mechanical spectrum of atenolol oral formulation after 180 days of storage at 25 °C.

After 180 days of storage at both temperatures, the mechanical spectrum of atenolol and enalapril maleate formulations did not suffer any relevant changes, which indicates that the inner structure of these structured fluids is preserved (Figure 12 and Figures S17–S19).

#### 2.2.4. Preservative Effectiveness

After 180 days of storage, the reduction in microbial count in all the microorganisms tested (*Escherichia coli* ATCC 8739, *Pseudomonas aeruginosa* ATCC 9027, *Staphylococcus aureus* ATCC 6538, *Candida albicans* ATCC 10231, and *Aspergillus brasiliensis* ATCC 16404) confirmed that the preservative system in atenolol and enalapril maleate formulations successfully prevented the growth of the challenge organisms, in accordance with USP Chapter <51> requirements, thus conferring the desired microbiological stability when stored either at 5 °C or 25 °C (Table 9 and Table S7) [44].

#### 2.2.5. Active Substance Assay

The HPLC-UV method was developed and validated for each drug, according to international guidelines to support the stability indicative assay. The chromatographic conditions were previously adjusted to clearly separate degradation products, while the use of two detectors (UV and fluorescence) further enhanced the method’s capacity to accurately quantity the unaltered drugs.

**Figure 13:**
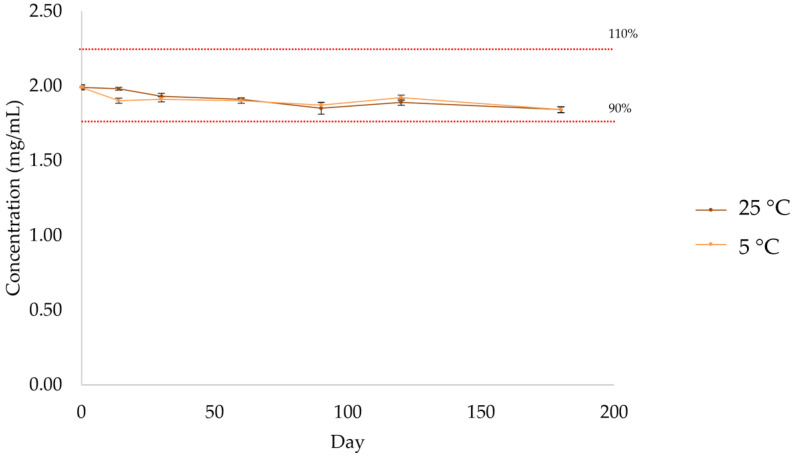
Quantification by HPLC-UV of atenolol in the formulation after storage at 5 °C and 25 °C.

The concentration of atenolol was above 90% during the entire assay, with similar concentrations under both storage conditions (Figure 13, Table 10 and Table S8). According to the US Pharmacopoeia, compounded preparations are considered to be stable if the drug concentration remains within 90–110% of the initial value (day 0).

**Figure 14:**
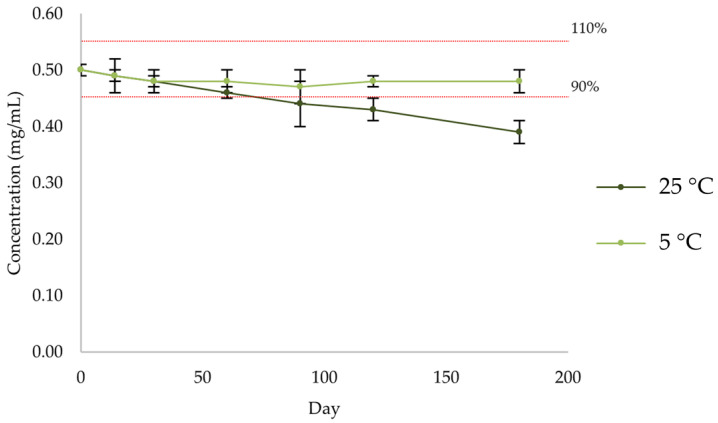
Quantification by HPLC-UV of enalapril maleate in the formulation after storage at 5 °C and 25 °C.

There was a progressive decrease in the amount of enalapril maleate (Figure 14, Table 11 and Table S9) in the formulation kept at room temperature (25 °C), while the amount of enalapril maleate in the formulation kept at refrigerated temperature (5 °C) remained within the 90–110% range. For the formulation stored at room temperature, the percentage of active substance decreased below 90% from day 90 onwards.

## 3. Discussion

Compounded medicines must meet specific quality standards to ensure safety and therapeutic effectiveness. Despite not requiring regulatory approval, pharmaceutical compounding is governed by national or state boards of pharmacy and must comply with USP compounding requirements or the European Pharmacopeia (EP) monograph regarding pharmaceutical preparations [1]. When developing compounded medicines, it is crucial to consider the harmful potential of excipients for the target population as well as their organoleptic properties and suitability for the intended type of administration, thereby potentiating patients’ acceptability and medication adherence.

The palatability of a pharmaceutical formulation refers to its level of pleasantness concerning several organoleptic properties, in particular taste [17,45]. Since unpalatable oral dosage forms can impact patients’ acceptability of the compounded medicine and medication adherence, palatability is a key parameter [46]. Therefore, the bitterness-blocking potential of the atenolol vehicle or SuspendIt^®^ vehicle has been verified through E-tongue assays. These assays showed that the bitterness of atenolol or enalapril maleate aqueous solutions could be effectively suppressed by dissolving these drugs in the oral vehicles under study. Previously, the research team successfully used a similar chemometric-sensor approach to evaluate the ability of various vehicles to mask the bitterness of oral solutions of ranitidine [17].

The mechanical properties of liquid dosage forms should allow easy administration, accurate measurement, and proper mixing and ensure adequate physical stability. Whenever desired, viscosity modifiers should be smartly selected to provide the desired rheological behavior. Using hydrophilic polymers as thickening agents is a common strategy to increase the viscosity of oral pharmaceuticals. In the case of SuspendIt^®^, amorphophallus konjac root powder and xanthan gum are synergistically combined to provide a three-dimensional structure that imparts a viscosity increase. This increase should be controlled to be within the desired range to facilitate all operations during product handling and administration and maintain physical stability [21]. Furthermore, while many proprietary oral vehicles are commonly classified as suspensions due to their high viscosity, the tested formulations in this study are solutions from a physical standpoint since atenolol and enalapril maleate are water-soluble at the concentrations used. The formulations are clear, without any opacity or residue. The atenolol and enalapril maleate formulations exhibited shear-thinning behavior, which means that their apparent viscosity decreases when subjected to stress. Polymers are characterized by an entanglement with a random orientation of the inner structure when at rest [21,47,48]. In shear-thinning behavior, when sufficient shear is applied, the polymer chains begin to disentangle [48]. Individual molecules exhibit less flow resistance than entangled structures, resulting in shear-thinning flow behavior with decreasing viscosity at higher shear rates [48]. Upon removal of the shear stress, the internal structure of the material gradually reassembles, resulting in the recovery of viscosity to its original level over time [48]. This time-dependent behavior is called thixotropy. The shear-thinning behavior shown by the atenolol and enalapril maleate formulations facilitates their administration orally and through enteral feeding tubes. The active substances are dissolved in the vehicle SuspendIt^®^, which minimizes any impact on the mechanical properties. Consequently, no differences in the type of flow behavior between the vehicle and the formulations were observed. Shear-thinning behavior is typical of polymeric dispersions used in pharmaceutical formulations such as konjac and xanthan gum (the thickeners used in SuspendIt^®^), carbomer, and poloxamer dispersions [49,50,51,52]. The formulations become more fluid upon shaking, thus allowing a convenient administration. Also, when injected through a feeding tube, progressive fluidification ensures an effortless administration. Yield stress, defined as the minimum shear stress required to initiate flow, is a helpful parameter to evaluate the product’s performance and processability and predict the product’s long-term stability and shelf life [45]. Yield stress reduces the ability to flow under shipping vibrations and gravity [53]. Since the existence of apparent yield stress indicates a more structured fluid, an influence on the swallowing process and mouthfeel is also expected [54]. Both liquid dosage forms presented an apparent yield stress, as described by the Heschel-Bulkley rheological model, thus reinforcing their suitability for oral administration and predicting good physical stability. The thixotropic behavior exhibited by the cardiovascular oral liquids implies their capacity to undergo thinning upon agitation and time-dependent recovery of the viscosity upon settling [55]. The recovery plays a pivotal role in ensuring enhanced physical stability of the formulations [55]. Also, as the recovery of the viscosity is not immediate, the formulations can easily be injected through the feeding tube. The tested formulations also presented viscoelastic behavior typical of polymeric dispersions [48]. Polymers are viscoelastic materials due to their ability to provide mechanical responses across different length scales, from short-range to long-range, when subjected to an applied force [56]. The amplitude sweep test stands as a relevant tool for understanding the dynamics of material behavior as it progresses from solid-like to liquid-like under increasing deformation [57]. This test offers insights into the flexibility and structural integrity of materials, thereby facilitating a comprehensive characterization of their viscoelastic properties [57]. The linear viscoelastic region (LVER) is obtained as the region wherein stress varies proportionally to deformation until a critical strain is attained [57]. The larger this region, the greater the material’s resistance to deformation. The critical strain was reached at around 10% for both formulations, which is similar in comparison with other oral liquids such as xanthan gum, guar gum, locust bean gum, and konjac dispersions [47,58,59], foreseeing a good resistance to deformation for the atenolol and enalapril maleate formulations. Below the critical strain, the storage modulus of the formulations was higher than the loss modulus. This behavior is consistent with that observed in other polymeric dispersions, such as xanthan gum [47]. The frequency sweep test is used to obtain the mechanical spectrum of a material that represents its “fingerprint” since it is unique to each material [60]. This test is performed in the LVER and is employed to investigate the viscoelastic properties of a material [60]. For both cardiovascular formulations, the storage modulus was higher than the loss modulus, implying a strong association between the hydrophilic polymers and a solid-like behavior in the measured frequency span. No marked differences were observed in the oscillatory measurements (amplitude and frequency sweep) between the formulations and the vehicle SuspendIt^®^. This is likely due to the molecular dissolution of the active substances in the vehicle, which does not affect the inner structure afforded by the thickeners. Taken together, the results of the mechanical properties of the cardiovascular formulations support their use for oral and enteral feeding tube administration, predicting good physical stability. The avoidance of potentially harmful excipients is recommended wherever possible in pediatric formulations, especially for neonates [61]. The proposed cardiovascular formulations are free of excipients such as parabens, sorbitol, propylene glycol, and benzyl alcohol. Vehicles used to compound oral liquid medications of many drugs reported in the literature usually contain parabens, since aqueous liquid formulations present a risk of microbiological instability [62,63,64].

Stability tests ensure that compounded medicines remain effective and safe for use throughout their intended shelf life, thereby providing accurate and reliable beyond-use dates. HPLC-UV methods were developed and validated for each drug, according to international guidelines. Both chromatographic systems were previously tested for their capacity to separate compounds resulting from forced degradation from the main peaks using drug standards submitted to acidic (HCl 1 M), basic (NaOH 0.1 M), heat (100 °C, 1 h), and light exposure (500 W, 1 h) conditions. Their stability-indicating capability was confirmed by verifying that degradation products (resulting from subjection to heat, acid and basic conditions, and oxidation) did not coelute with the drugs. These stability-indicating methods are important to properly investigate chemical stability over time, especially for pediatric patients, because they have immature organs, slower metabolism, and elimination capabilities and are more susceptible to the deleterious consequences that may arise from the presence of harmful degradation products [65]. Regarding the organoleptic characteristics, a yellowish darkening occurred over time in both formulations when stored at 25 °C. The same happened with SuspendIt^®^, which may suggest that this color change is inherent to the vehicle itself and not due to the degradation of the active substances. On the contrary, refrigeration preserved the original light color for all preparations. Based on the appearance of the formulations, no apparent precipitation of the drugs was observed over the storage period. It would also be important to test the formulations in biorelevant media, such as FaSSIF and FeSSIF, to determine if any drug precipitation occurs, as this is essential to ensure oral bioavailability.

The stability-indicating assay suggested the feasibility of using liquid dosage forms of atenolol (stored at both room and refrigerated temperatures) and enalapril maleate (stored at refrigerated temperature) with SuspendIt^®^ within a 180-day period after preparation. The only exception was the enalapril maleate preparation stored at room temperature, which showed a decrease in drug content below 90% from 90 days onwards. Therefore, compounding pharmacists can recommend patients and healthcare professionals store enalapril maleate formulation in a refrigerator to ensure optimal preservation. Significant variations in pH notably impact the solubility and stability of drugs in formulations, as they can reflect the chemical alteration of the compounded medicines, underscoring the importance of monitoring this parameter over time [66]. During the 180-day storage period, the pH of the atenolol and enalapril maleate formulations showed negligible variation, suggesting that no marked chemical alterations occurred. The shear-thinning behavior of the formulation was maintained over the 180 days of analysis for both room and refrigerated storage temperatures. Thixotropic behavior was also maintained over time, with slight variations in recovery rate and recovery time for both oral formulations at both temperatures. The LVER of the oral formulations did not change over the 180-day storage period, and the critical strain remained around 10%. The characteristic mechanical spectrum of both formulations at different storage temperatures also did not change over the analysis time. The observed mechanical stability confirmed the prediction of good stability resulting from the rheological analysis previously performed. 

The evaluation of the preservative effectiveness, following USP Chapter <51> requirements, confirmed that the preservative system in atenolol and enalapril maleate remained effective throughout the storage period, either at 5 °C or 25 °C. The preservative systems successfully inhibited the growth of challenge microorganisms, both bacteria and fungus, which is particularly relevant in multidose aqueous liquid formulations that present a higher risk of microbiological contamination. This result reflects that the preservative system is adequate for these preparations, ensuring microbiological quality, safety, and extended shelf life over analysis time.

The poor stability of atenolol and enalapril maleate in aqueous solutions presents a notable obstacle to the creation of liquid dosage forms [67,68,69]. Degradation was observed as soon as 30 min for enalapril maleate [70] and may be influenced by pH changes as well as by the water sorption activity of excipients in contact with this active substance [71]. The degradation of atenolol in an aqueous solution is also affected by pH variations, with increased stability observed at lower (acidic) pH levels [72]. Consequently, there is a need to develop oral vehicles capable of preserving the stability of these active substances while masking the bitterness characteristic of atenolol and enalapril maleate [73,74]. Previous studies on the chemical stability of atenolol and enalapril maleate in other oral vehicles reported beyond-use dates between 30–90 days for enalapril and 9 to 40 days for atenolol [67,69,75,76,77]. In this work, the use of SuspendIt^®^ as a vehicle for atenolol and enalapril maleate, stored under refrigerated conditions, led to an extended shelf life of the products to 180 days, which is a result much greater than the aforementioned stability studies.

## 4. Materials and Methods

### 4.1. Chemicals and Reagents

SuspendIt^®^, atenolol USP, enalapril maleate USP, citric acid USP, sodium citrate USP, acesulfame, and steviol glycosides (95%) were provided by the Professional Compounding Centers of America (PCCA) (Houston, TX, USA). Sucrose was obtained from Acofarma (Barcelona, Spain). Lyophilized microorganisms, *Escherichia coli* ATCC 8739, *Pseudomonas aeruginosa* ATCC 9027, *Staphylococcus aureus* ATCC 6538, *Candida albicans* ATCC 10231, and *Aspergillus brasiliensis* ATCC 16404, were obtained from Liofilchem (ATCC licensed derivatives) by Frilabo (Maia, Portugal). Tryptic Soy Agar (TSA) and Sabouraud Dextrose Agar culture mediums were also obtained from Frilabo (Maia, Portugal).

The simple syrup is a 66.7% aqueous sucrose solution prepared according to the Portuguese Galenic Form using 66.7% (*w*/*w*) sucrose and 33.3% (*w*/*w*) purified water, and it was used in the injectability test [43]. The sucrose was weighed and added to the purified water while being stirred manually. The mixture was heated in a water bath at a temperature of 70–80 °C to help dissolve the sucrose and stirred throughout the process. Finally, sufficient purified water, previously heated to 50 °C, was added to complete the volume, and then the solution was filtered through a Chardin paper filter.

The oral formulations of atenolol (Table 12) and enalapril maleate (Table 13) in SuspendIt^®^ were prepared according to formulations developed by the supplier of SuspendIt^®^ (PCCA). The atenolol vehicle corresponds to SuspendIt^®^ with additional excipients. For the atenolol formulation, atenolol, citric acid monhydrate, sodium citrate dihydrate, acesulfame potassium, and steviol glycosides (95%) were triturated to reduce particle size. SuspendIt^®^ was then added until it formed a smooth paste. More SuspendIt^®^ was added in portions with magnetic stirring for 20 min. Finally, the volume of the atenolol formulation was topped up with SuspendIt^®^. For the enalapril maleate formulation, SuspendIt^®^ and enalapril maleate were mixed with magnetic stirring for 20 min. Finally, the volume of the enalapril maleate formulation was topped up with SuspendIt^®^.

### 4.2. Preliminary Characterization of SuspendIt^®^ Vehicle and the Oral Suspensions

A comparison study was carried out between the vehicle used—SuspendIt^®^—and the atenolol and enalapril maleate oral formulations to be studied. To this purpose, pH, rheological properties, injectability, and palatability were analyzed as described below. 

#### 4.2.1. pH

The pH was measured in triplicate on a HI 2211 pH/ORP/°C meter (Hanna Instruments, Woonsocket, RI, USA) at 25 °C.

#### 4.2.2. Rheological Properties

The rheological properties (flow curve, thixotropy, linear viscoelastic region, and mechanical spectrum) were determined on a Kinexus Prime lab+ Rheometer (Malvern Panalytical, Malvern, UK) at 25 °C. The cone and plate geometry (cone angle: 4°; diameter: 40 mm; working gap: 0.150 mm) was used in all tests. For the determination of the flow curve, measurements were performed in the shear rate range of 0.1 s^−1^ to 100 s^−1^. A rheological model was fitted to the results, namely the Herschel-Bulkley model. This model is employed to characterize the rheological behavior of non-Newtonian fluids, where the stress-strain relationship is nonlinear [78]. This model incorporates a yield stress parameter and a power law index, effectively capturing the shear-thinning properties of these fluids [78]. The shear rate induced by a specific shear stress applied to the fluid can be quantified using the following equation:

$$\tau =\tau_{0}+K{\left(\frac{du}{dy}\right)}^{n}$$

where du/dy is the shear rate, τ is the shear stress, τ_0_ is the yield stress, K is the consistency coefficient, and n is the flow index.

For the determination of the linear viscoelastic region, an amplitude sweep was carried out in the shear strain range of 0.1% to 100% at a frequency of 1.0 Hz. For the mechanical spectrum, a frequency sweep was carried out from 10 to 0.1 Hz. at a shear strain of 0.4%. To assess the thixotropic behavior, a three-step test was performed under the following conditions: shear rate, 0.1 s^−1^; time in the first phase, 1 min; shear rate, 100 s^−1^; time in the second phase, 30 s; shear rate: 0.1 s^−1^; time in the third phase, 10 min. All measurements were obtained in triplicate. 

#### 4.2.3. Injectability

**Figure 15:**
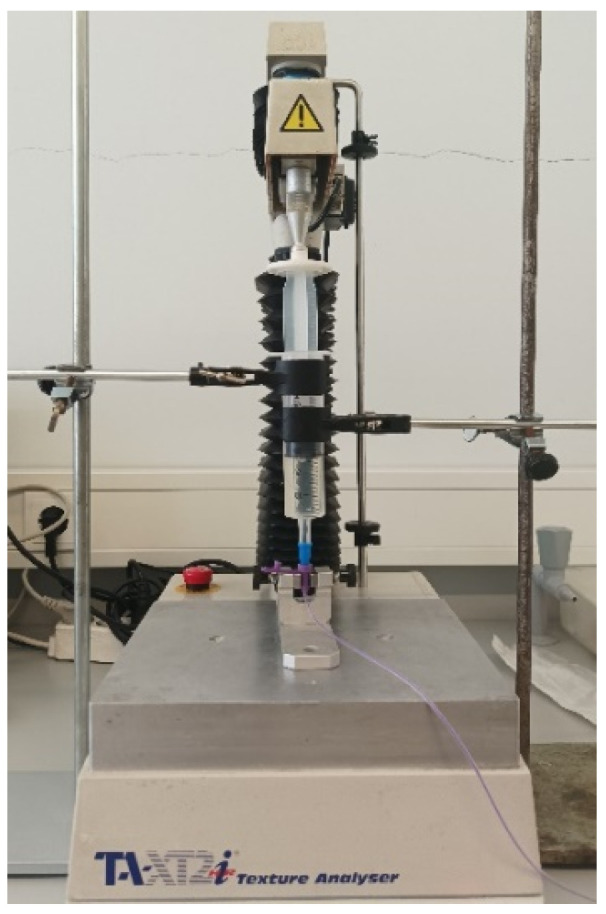
Injectability test set up in the texture analyzer TA.XT.plus (Stable Micro Systems, Godalming, UK).

The injectability test was performed using a texture analyzer TA.XT.plus (Stable Micro Systems, Godalming, UK) at 25 °C (Figure 15). The injectability test aims to measure the force required to promote the flow of the oral suspensions through a 50 mL syringe coupled to NeoMed^®®^ Radiopaque, Polyurethane (PUR)—Enteral Feeding Tube with Connector (outer diameter: 6.5FR; length: 90 cm). The syringe was filled using with the sample (30 mL), and the whole device was mounted on the texture analyzer working in compression mode. During extrusion tests, samples were assayed in triplicate, applying the force vertically and using a plunger displacement rate of 0.2 mm/s for a distance of 25 mm. The simple syrup was also tested for comparison.

#### 4.2.4. E-tongue Apparatus and Potentiometric Analysis of Atenolol and Enalapril Maleate Solutions

A custom-designed potentiometric E-tongue with 40 lipid polymeric sensor membranes was employed as a taste-sensing tool to assess the ability of the atenolol vehicle and the SuspendIt^®^ vehicle to mask bitterness in atenolol and enalapril maleate formulations. The specifications of the E-tongue device, number of sensor arrays, sensor membrane types, and data acquisition apparatus and software were previously outlined in a study by the research team [17]. For the experimental procedures, six independent samples (100 mL) of each oral formulation were prepared: atenolol in aqueous solution (2 mg/mL), atenolol in atenolol vehicle (2 mg/mL), enalapril maleate in aqueous solution (0.5 mg/mL), and enalapril maleate in SuspendIt^®^ vehicle (0.5 mg/mL). Additionally, six independent 100 mL samples of each pure vehicle (without any drug) were also prepared. Due to the challenge of conducting potentiometric assays in non-conductive liquids with high viscosity, the initial solutions were diluted ten times with deionized water to facilitate electrochemical analysis and prevent damage to the lipid sensor membranes of the E-tongue. The solutions were analyzed at ambient temperature (≈20 °C) using the E-tongue. Each analysis lasted 5 min, allowing for a pseudo-equilibrium to be reached between the E-tongue’s non-specific lipid polymeric membranes and the constituents of each solution. Following each assay, the E-tongue was rinsed with deionized water, and after four to five assays, it was submerged in a 0.01 mol/L HCl aqueous solution to assess signal repeatability and ensure effective cleaning of the sensor arrays. At the conclusion of each day, the E-tongue was stored at room temperature and immersed in a 0.01 mol/L HCl cleaning solution to maintain the integrity and functionality of the lipid polymeric membranes over extended periods.

##### Statistical Analysis

The discrimination capability of the E-tongue was evaluated using chemometric techniques. Specifically, Linear Discriminant Analysis (LDA) combined with the simulated annealing (SA) variable selection algorithm, as detailed previously [17], was employed. This algorithm enabled the identification of the most pertinent subset of non-redundant E-tongue sensors. Assessment of LDA performance was conducted on both the original grouped data (training) and through leave-one-out cross-validation (LOO-CV). The visualization of the former involved 2D plots of the two most influential discriminant functions (DFs), while the latter was evaluated based on sensitivity values, representing the percentage of accurately classified samples within predetermined groups. LOO-CV, commonly used for small datasets lacking an external validation dataset, was employed in this study. All statistical analyses were performed using the packages of the open-source statistical software *R* (RStudio 2024.04.0 Build 735), at a 5% significance level.

### 4.3. Stability Study

Afterwards, a six-month stability study was conducted for atenolol and enalapril maleate oral formulations prepared with SuspendIt^®^ by evaluating the chemical, physical, and microbiological stability. Three independent batches (1.7 L each) of the oral suspensions of atenolol and enalapril maleate were prepared. Samples were stored in amber plastic prescription bottles at two temperature conditions (5 °C and 25 °C). Samples were assayed initially and at pre-determined time intervals over a six-month period. Together with the drug assays, physicochemical properties such as pH and rheological behavior were measured. These measurements were performed initially and after 14, 30, 60, 90, 120, and 180 days of storage. Additionally, formulations were tested for microbiological stability, namely the preservative effectiveness test (initially and after 30, 90, and 180 days of storage), according to USP Chapter <51> antimicrobial effectiveness testing [44]. The appearance and odor of the formulations were also recorded over time. The odor analysis was conducted using olfactory sensory evaluation by a human assessor. Although small changes can go undetected, marked odor modification is likely to be perceived [79].

#### 4.3.1. Preservative Effectiveness

SuspendIt^®^ contains potassium sorbate and sodium benzoate as antimicrobial preservatives. The standard challenge organisms described in the USP (USP Chapter <51> antimicrobial effectiveness testing includes *Escherichia coli* ATCC 8739, *Pseudomonas aeruginosa* ATCC 9027, *Staphylococcus aureus* ATCC 6538, *Candida albicans* ATCC 10231, and *Aspergillus brasiliensis* ATCC 16404 [44]. This test provides the following criteria to determine antimicrobial effectiveness:

- Bacteria: not less than 1 log reduction from the initial count at 14 days, and no increase from the 14 days’ count at 28 days;
- Yeasts and molds: no increase from the initial calculated count at 14 days and 28 days.

A suitability test of the counting method in the presence of the products was performed for each product as described in USP Chapter <51>. The suitability test is intended to verify the capacity of microorganisms to recover 50% growth compared with saline control. The products with no dilution, with one dilution (1 mL of product without dilution in 9 mL of sterile saline), and with two dilutions (1 mL of product with one dilution in 9 mL of sterile saline) were tested. Since the inhibitory capacity of the preservatives was very pronounced, preventing counting, it was necessary to dilute the products. For all the microorganisms, except for *P. aeruginosa*, one dilution (1 mL of product in 9 mL of sterile saline) of the products was needed.

#### 4.3.2. Active Substance Assay

Quantification of atenolol and enalapril maleate was performed using high-performance liquid chromatography (HPLC). The method was developed based on both EU and US Pharmacopeias, with minor adjustments, particularly with the inclusion of a precipitation step during sample preparation to avoid clogging the chromatographic column, and gradient adjustments to grant complete separation from matrix components. On each sampling day, the flasks for analysis were delivered to the HPLC lab. The vials were left at room temperature, and all samples and standard solutions were processed as a batch. The sample preparation procedure was also applied to all standard solutions. 

Briefly, 750 µL of homogenized preparations were transferred to Eppendorf tubes, followed by the addition of 500 µL of acetonitrile. After a brief vortexing with visual confirmation of precipitation, the tubes were centrifuged at 13,000 rpm for 5 min at room temperature. A 500-µL portion was transferred to the injection vials containing 1000 µL of buffer (monobasic sodium phosphate buffer prepared with 2.8 g per liter of water and pH adjusted to 2.5 with phosphoric acid). After homogenization, the vials were ready for injection. Under this procedure, the samples and standards were injected with a 5-fold dilution in comparison to their original concentration, corresponding to 0.1 mg/mL for enalapril and 0.4 mg/mL for atenolol preparations. This final dilution is equivalent to the one described in monographs.

Quantification was based on external calibration curves, with standards prepared daily in deionized water at 4.0 mg/mL for atenolol and at 1.0 mg/mL for enalapril, with dilutions in the working range (25 to 150% of the expected concentrations). Both water-based and matrix-based (SuspendIt^®^) working standards calibration curves were previously tested, with no differences between the two approaches. In addition to UV detection (228 nm for atenolol and 215 nm for enalapril), a second channel with fluorescence detection was included that, due to its specificity, allowed us to confirm the results obtained.

Table S1 resumes the HPLC method characteristics for both quantifications, including column, gradient, injection, etc. The methods were optimized to grant no coelution with other sample components, with the shortest analysis time, providing stable elution conditions, and with the lowest consumption of solvents due to our green-chemistry policy.

Both methods were validated to determine their adequacy and stability to support a 6-month study (Table S2). The tests included a definition of an adequate working range, repeatability over consecutive injections and consecutive extractions, as well as its accuracy testing of three spiking levels within the working range (75%, 100%, and 125%) and presenting its average.

## 5. Conclusions

This study underscored the comprehensive characterization needed when selecting a suitable vehicle for compounded liquid dosage forms suitable for oral and enteral feeding tube administration. For this purpose, palatability, injectability, rheological properties, pH, and physical, chemical, and microbiological stability were studied. Both cardiovascular formulations were found suitable for administration through enteral feeding tubes. Injectability tests showed both formulations required less force for injection through a syringe coupled to an enteral feeding tube compared to simple syrup. Moreover, the bitterness of drugs could be effectively masked by using the atenolol vehicle or SuspendIt^®^ vehicle, as confirmed by the taste sensor device, thus supporting adequate palatability and predicting good medication adherence. These results should, however, confirmed with the target patients. While both atenolol and enalapril maleate preparations showed overall stability in terms of mechanical properties and pH during the 180 days of storage at both temperatures, chemical stability presented a different scenario. A decrease in drug content below 90% was observed for enalapril maleate from 90 days onwards when stored at 25 °C. The effectiveness of the preservative system was maintained throughout the storage period, as per USP Chapter <51> requirements. In summary, the atenolol oral formulation maintained suitable chemical, physical, and microbiological stability for 180 days of storage at 5 °C and 25 °C, while enalapril maleate remained stable up to 60 days at 25 °C and for 180 days at 5 °C. The use of the proposed oral compounding formulations provides a customized, reliable, and less costly solution for the long-term treatment of cardiovascular patients.

## Figures and Tables

**Table 1:** Hershel-Bulkley model parameters for atenolol and enalapril maleate formulations and SuspendIt^®^ vehicle (mean ± standard deviation, *n = 3*).

Sample	K (Pa.s^n^)	n	τ_0_ (Pa)	R^2^
Atenolol formulation	0.238 ± 0.061	0.633 ± 0.030	0.756 ± 0.042	0.999 ± 0.000
Enalapril Maleate formulation	0.273 ± 0.028	0.594 ± 0.027	0.980 ± 0.068	0.998 ± 0.000
SuspendIt^®^	0.483 ± 0.007	0.516 ± 0.003	0.954 ± 0.003	0.999 ± 0.001

**Table 2:** Recovery period (50%) and recovery rate of SuspendIt^®^, atenolol, and enalapril maleate formulations (mean ± standard deviation, *n = 3*).

Sample	Recovery Period (s)	Recovery Rate (%)
Atenolol formulation	140.00 ± 13.64	69.12 ± 2.34
Enalapril Maleate formulation	150.56 ± 17.10	64.10 ± 6.99
SuspendIt^®^	120.0 ± 13.5	78.4 ± 3.1

**Table 3:** Mean force and standard deviation of SuspendIt^®^, simple syrup, USP vehicle, atenolol, and enalapril maleate formulations (*n = 3*).

Sample	Mean Force ± Standard Deviation (N)
Atenolol formulation	22.5 ± 0.2
Enalapril Maleate formulation	21.6 ± 1.5
Simple syrup	146.8 ± 2.0
SuspendIt^®^	22.8 ± 1.4

**Table 4:** pH of atenolol and enalapril maleate formulations (mean ± standard deviation, *n = 3*).

	Atenolol Formulation		Enalapril Maleate Formulation	
	5 °C	25 °C	5 °C	25 °C
Day 0	4.65 ± 0.00		4.95 ± 0.01	
Day 14	4.67 ± 0.01	4.66 ± 0.03	4.97 ± 0.01	4.95 ± 0.01
Day 30	4.73 ± 0.01	4.71 ± 0.01	4.95 ± 0.01	4.96 ± 0.01
Day 60	4.73 ± 0.01	4.74 ± 0.00	4.93 ± 0.01	4.98 ± 0.02
Day 90	4.69 ± 0.02	4.67 ± 0.02	4.90 ± 0.02	4.94 ± 0.06
Day 120	4.76 ± 0.02	4.76 ± 0.02	4.96 ± 0.01	4.98 ± 0.02
Day 180	4.72 ± 0.00	4.74 ± 0.02	4.94 ± 0.02	4.93 ± 0.03

**Table 5:** Hershel-Bulkley model parameters for atenolol formulation after storage at 25 °C (mean ± standard deviation, *n = 3*).

Day	0	14	30	60	90	120	180
K (Pa.s^n^)	0.238 ± 0.061	0.245 ± 0.031	0.257 ± 0.017	0.255 ± 0.055	0.221 ± 0.019	0.221 ± 0.053	0.239 ± 0.052
n	0.633 ± 0.030	0.629 ± 0.023	0.620 ± 0.004	0.618 ± 0.032	0.609 ± 0.023	0.608 ± 0.035	0.584 ± 0.048
τ_0_ (Pa)	0.756 ± 0.042	0.811 ± 0.088	0.763 ± 0.009	0.835 ± 0.050	0.786 ± 0.085	0.822 ± 0.098	0.827 ± 0.100
R^2^	0.999 ± 0.000	0.999 ± 0.001	1.000 ± 0.000	0.999 ± 0.001	0.999 ± 0.001	0.997 ± 0.002	0.995 ± 0.005

**Table 6:** Hershel-Bulkley model parameters for atenolol formulation after storage at 5 °C (mean ± standard deviation, *n = 3*).

Day	14	30	60	90	120	180
K (Pa.s^n^)	0.241 ± 0.037	0.239 ± 0.032	0.225 ± 0.038	0.247 ± 0.007	0.229 ± 0.024	0.221 ± 0.011
N	0.635 ± 0.034	0.639 ± 0.022	0.645 ± 0.025	0.611 ± 0.008	0.628 ± 0.025	0.613 ± 0.012
τ_0_ (Pa)	0.817 ± 0.112	0.821 ± 0.068	0.857 ± 0.050	0.767 ± 0.036	0.826 ± 0.083	0.738 ± 0.050
R^2^	0.999 ± 0.000	0.999 ± 0.000	0.999 ± 0.000	0.999 ± 0.000	0.999 ± 0.000	0.999 ± 0.000

**Table 7:** Hershel-Bulkley model parameters for enalapril maleate formulation after storage at 25 °C (mean ± standard deviation, *n = 3*).

Day	0	14	30	60	90	120	180
K (Pa.s^n^)	0.273 ± 0.028	0.298 ± 0.030	0.290 ± 0.075	0.265 ± 0.019	0.210 ± 0.040	0.216 ± 0.040	0.229 ± 0.002
n	0.594 ± 0.027	0.586 ± 0.018	0.584 ± 0.048	0.606 ± 0.014	0.614 ± 0.027	0.635 ± 0.036	0.586 ± 0.007
τ_0_ (Pa)	0.980 ± 0.068	1.086 ± 0.015	1.076 ± 0.052	1.116 ± 0.030	1.077 ± 0.038	1.209 ± 0.068	1.128 ± 0.089
R^2^	0.998 ± 0.000	0.995 ± 0.002	0.997 ± 0.001	0.996 ± 0.004	0.993 ± 0.000	0.988 ± 0.005	0.985 ± 0.009

**Table 8:** Hershel-Bulkley model parameters for enalapril maleate formulation after storage at 5 °C (mean ± standard deviation, *n = 3*).

Day	14	30	60	90	120	180
K (Pa.s^n^)	0.226 ± 0.035	0.250 ± 0.118	0.203 ± 0.017	0.226 ± 0.029	0.262 ± 0.022	0.192 ± 0.026
N	0.641 ± 0.029	0.592 ± 0.064	0.655 ± 0.016	0.621 ± 0.022	0.593 ± 0.009	0.641 ± 0.024
τ_0_ (Pa)	1.115 ± 0.069	1.012 ± 0.092	1.143 ± 0.062	1.063 ± 0.026	1.003 ± 0.044	1.113 ± 0.107
R^2^	0.998 ± 0.001	0.999 ± 0.001	0.997 ± 0.001	0.997 ± 0.002	0.998 ± 0.001	0.993 ± 0.006

**Table 9:** United States Pharmacopeia Chapter <51> Antimicrobial Effectiveness Test of atenolol oral formulation.

Challenge Microorganism	Day	5 °C	25 °C
*Escherichia coli*	0	Pass *	
*Pseudomonas aeruginosa*		Pass **	
*Staphylococcus aureus*		Pass *	
*Candida albicans*		Pass **	
*Aspergillus brasiliensis*		Pass **	
*Escherichia coli*	30	Pass *	Pass *
*Pseudomonas aeruginosa*		Pass *	Pass *
*Staphylococcus aureus*		Pass *	Pass *
*Candida albicans*		Pass *	Pass *
*Aspergillus brasiliensis*		Pass *	Pass *
*Escherichia coli*	90	Pass *	Pass *
*Pseudomonas aeruginosa*		Pass *	Pass *
*Staphylococcus aureus*		Pass *	Pass *
*Candida albicans*		Pass *	Pass *
*Aspergillus brasiliensis*		Pass *	Pass *
*Escherichia coli*	180	Pass *	Pass *
*Pseudomonas aeruginosa*		Pass *	Pass *
*Staphylococcus aureus*		Pass *	Pass *
*Candida albicans*		Pass *	Pass *
*Aspergillus brasiliensis*		Pass *	Pass *

* >1 log reduction after 14 days; ** 1 log reduction after 14 days.

**Table 10:** Quantification of atenolol by HPLC-UV after storage at room temperature (*n = 3*, 25 °C).

Day	0	14	30	60	90	120	180
Mean (mg/mL)	1.99	1.98	1.93	1.91	1.85	1.89	1.84
SD	0.01	0.01	0.02	0.01	0.04	0.02	0.02
%	99	99	97	95	93	95	92

**Table 11:** Quantification of enalapril by HPLC-UV after storage at room temperature (*n = 3*, 25 °C).

Day	0	14	30	60	90	120	180
Mean (mg/mL)	0.50	0.49	0.48	0.46	0.44	0.43	0.39
SD	0.01	0.00	0.01	0.00	0.00	0.00	0.01
%	101	97	96	91	88	85	78

**Table 12:** Atenolol 2 mg/mL oral formulation composition.

	Atenolol USP	Active Substance	0.200 g
Atenolol vehicle	Citric acid USP monohydrate	pH buffer/chelating agent	0.935 g
	Sodium citrate dihydrate USP	pH buffer/chelating agent	1.632 g
	Acesulfame potassium FCC	Sweetener	0.100 g
	Steviol glycosides 95%	Sweetener	0.100 g
	SuspendIt^®^	Suspending agent	q.s. 100 mL

**Table 13:** Enalapril Maleate 0.5 mg/mL oral formulation composition.

Enalapril Maleate USP	Active Substance	0.05 g
SuspendIt^®^	Suspending agent	q.s. 100 mL

## Data Availability

Data is contained within the article and Supplementary Materials.

## Supplementary Materials

The following supporting information can be downloaded at: https://www.mdpi.com/article/10.3390/ph17081052/s1, Figure S1: Overlaid chromatograms from standard solutions prepared in HPLC water from a calibration curve prepared for atenolol (A) and enalapril (B) analysis; Figure S2: Chromatogram from an enalapril standard solution detected under UV and fluorescence detection.; Figure S3: Chromatograms from six atenolol samples at T90: atenolol eluting at 4.4 min, completely separated from the other matrix components under UV and FLD detections.; Figure S4: Chromatograms from a triplicate set of enalapril samples analyzed at day 30, with the UV reading above and the FLD below.; Figure S5: Overlay chromatograms from the degradation studies, with the UV reading above and the FLD below; Figure S6: Appearance of SuspendIt^®^ (left) and atenolol (middle) and enalapril maleate (right) formulations after 180 days storage at 25 °C; Figure S7: Appearance of SuspendIt^®^ (left) and atenolol (middle) and enalapril maleate (right) formulations after 180 days storage at 5 °C; Figure S8: Flow curve of atenolol oral formulation after storage at 5 °C; Figure S9: Flow curve of enalapril maleate oral formulation after storage at 25 °C; Figure S10: Flow curve of enalapril maleate oral formulation after storage at 5 °C; Figure S11: Thixotropic behavior of atenolol oral formulation after storage at 5 °C; Figure S12: Thixotropic behavior of enalapril maleate oral formulation after storage at 25 °C; Figure S13: Thixotropic behavior of enalapril maleate oral formulation after storage at 5 °C; Figure S14: Amplitude sweep for atenolol oral formulation after 180 days storage at 5 °C; Figure S15: Amplitude sweep for enalapril maleate oral formulation after 180 days storage at 25 °C; Figure S16: Amplitude sweep for enalapril maleate oral formulation after 180 days storage at 5 °C; Figure S17: Mechanical spectrum of atenolol oral formulation after 180 days storage at 5 °C; Figure S18: Mechanical spectrum of enalapril maleate oral formulation after 180 days storage at 25 °C; Figure S19: Mechanical spectrum of enalapril maleate oral formulation after 180 days storage at 5 °C. Table S1: HPLC method characteristics.; Table S2: Overall performance of the analytical methods; Table S3: Recovery period (s) (50%) of atenolol formulation (mean ± standard deviation).; Table S4: Recovery rate (%) of atenolol formulation (mean ± standard deviation).; Table S5: Recovery period (s) (50%) of enalapril maleate formulation (mean ± standard deviation).; Table S6: Recovery rate (%) of enalapril maleate oral formulation (mean ± standard deviation).; Table S7: United States Pharmacopeia Chapter <51> Antimicrobial Effectiveness Test of enalapril maleate oral formulation; Table S8: Quantification of atenolol by HPLC-UV after storage at refrigerated temperature (n = 3, 5 °C).; Table S9: Quantification of enalapril by HPLC-UV after at refrigerated temperature (n = 3, 5 °C).
