# Supplementary material for: Characterization of Liquid Dosage Forms of Atenolol and Enalapril Maleate for Oral and Enteral Feeding Administration
Source: Pharmaceuticals (Basel). 2024 Aug 9;17(8):1052. doi: 10.3390/ph17081052 (PMC11359931; doi:10.3390/ph17081052)
Supplement: Supplementary file 1 [file pharmaceuticals-17-01052-s001.zip › pharmaceuticals-3069189-supplementary.pdf]

Supplementary material for:

Characterization of Liquid Dosage Forms of Atenolol and Enalapril Maleate for Oral and Enteral Feeding Administration

Sandra Mota[1,2], Ana Torres[1,2], Clara Quintas[2,3], António M. Peres[4], Nuno Ferreiro[4], Rebeca Cruz[5], Helena Ferreira[2,6,*], Isabel F. Almeida[1,2,*] and Susana Casal[5]

[1] Associate Laboratory i4HB - Institute for Health and Bioeconomy, University of Porto, 4050-313 Porto, Portugal; up201608486@up.pt (S.M.); up201706122@up.pt (A.T.); claraquintas@ff.up.pt (C.Q.)
[2] UCIBIO – Applied Molecular Biosciences Unit, Faculty of Pharmacy, Department of Drug Sciences, University of Porto, 4050-313 Porto, Portugal
[3] UCIBIO—Applied Molecular Biosciences Unit, Faculty of Pharmacy, Department of Drug Sciences, Laboratory of Pharmacology, University of Porto, 4050-313 Porto, Portugal
[4] CIMO, SusTEC, Instituto Politécnico de Bragança, Bragança, Portugal; peres@ipb.pt (A.M.P.); nuno.ferreiro@ipb.pt (N.F.)
[5] Associated Laboratory for Green Chemistry (LAQV) of the Network of Chemistry and Technology (REQUIMTE), Department of Chemical Sciences, Faculty of Pharmacy, Laboratory of Bromatology and Hydrology, University of Porto, 4050-313 Porto, Portugal; rcruz@ff.up.pt (R.C.); sucasal@ff.up.pt (S.C)
[6] UCIBIO–Applied Molecular Biosciences Laboratory of Microbiology, Department of Biological Sciences, Faculty of Pharmacy, University of Porto, 4050-313 Porto, Portugal

Correspondence: ifalmeida@ff.up.pt (I.F.A.), hferr@ff.up.pt (H.F.)

2. Materials and Methods

Table S1. *HPLC method characteristics*

	Atenolol	Enalapril
**Column**	Gemini 5 µm NX-C18 110 Å; 150 x 4.6 mm (Phenomenex)	
**Equipment**	JASCO (Japan)	
**Detection**	UV 228 nm; FLD 276/296 nm	UV: 215 nm FLD: 260/284 nm
**Eluents**	eluent A: 950 ml of buffer + 50 ml of acetonitrile<br>eluent B: 340 ml of buffer + 660 ml of acetonitrile	
**Gradient**	97%(A)/3%(B) until 5'; 1%/99% at 7'; 97%/3% at 8´	90%(A)/10%(B); 10%/90% from 3 to 10'; 90%/10% at 10.5'
**Run time**	18 min	20 min
**Flow rate**	1 ml/min	1 ml/min
**Temperature**	20 °C	55 °C
**Injection volume**	5 microliters	30 microliters

Table S2. *Overall performance of the analytical methods*

	Atenolol	Enalapril
**Retention time**	» 4.2 min	» 4.4 min
**LOD**	0.005 mg/ml	0.001 mg/ml
**LOQ**	0.01 mg/ml	0.003 mg/ml
**Working range**	0.1- 4 mg/ml	0.1-2 mg/ml
**Correlation coefficient**	>0.998	>0.998
**Repeatability injection**	standards (n=5) - RT = 0.2%<br>sample (n=3) – RT = 0.2%	standards (n=5) – RT = 0.05%<br>sample (n=3) – RT = 0.1%
**Repeatability extraction**	Sample (n=3) < 0.6%	Sample (n=3) < 0.8%
**Accuracy**	100 ± 2 %	100 ± 2 %

The injection repeatability corresponds to the coefficient of variation of the retention time between five consecutive injections of standards or sample solutions, showing excellent stability of the HPLC injection system (<0.2%). The repeatability of extraction corresponds to the variation in the quantification of a sample extracted three times over the same day, using the same calibration curve, also with <1% on both methods. The limit of detection and quantification (LOD and LOQ) were calculated on a signal-to-noise ratio of 3:1 and 10:1, respectively. Accuracy was tested by spiking with the drug at three different levels, followed by the complete extractive procedure, and calculated as the percentage deviation from the theoretical concentrations.

Fig. S1 represents overlayed chromatograms of atenolol (S1A) and enalapril maleate (S1B) under UV detection from standard solutions from calibration curves. Atenolol standards provided clean chromatograms, with a geometric peak at 4.2 minutes while enalapril showed two peaks,

corresponding to maleate and enalapril, the latter eluting at 4.4 min under the established conditions.

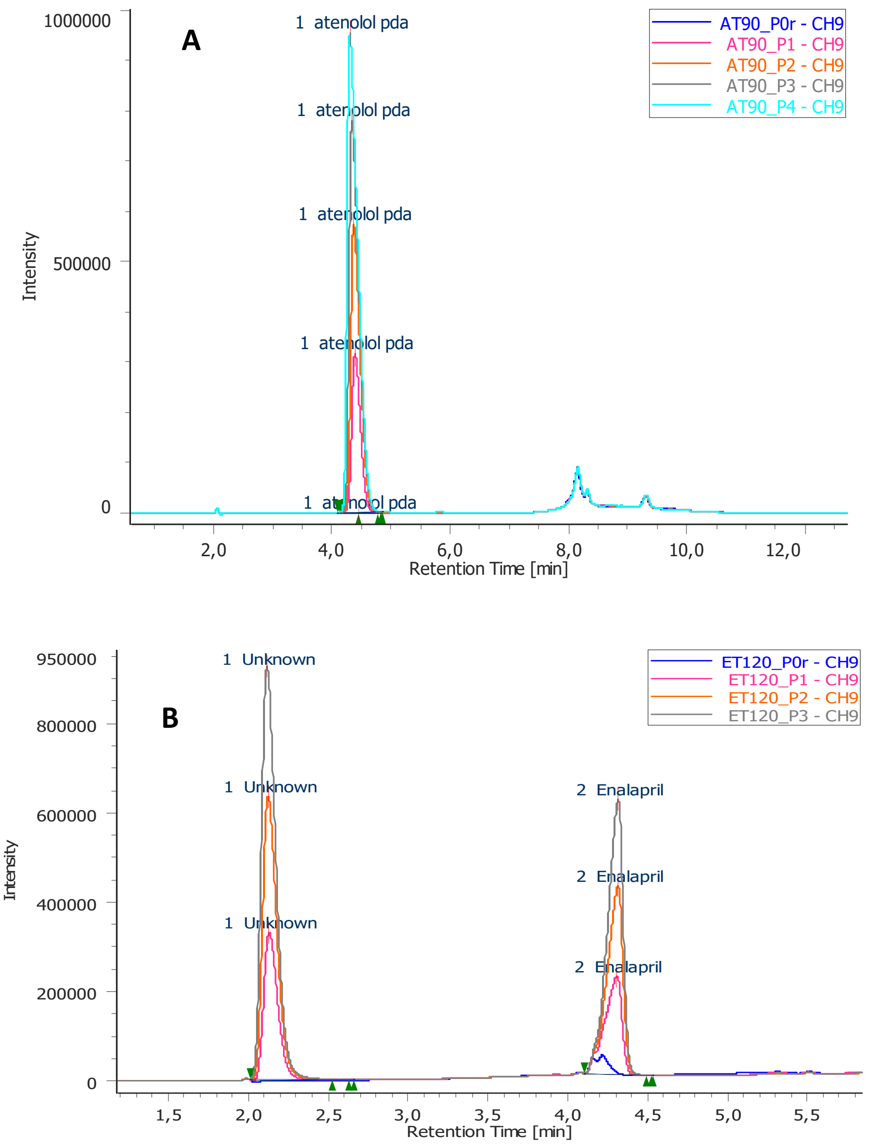

Figure S1. *Overlaid chromatograms from standard solutions prepared in HPLC water from a calibration curve prepared for atenolol (A) and enalapril maleate (B) analysis*

By complementing detection with the fluorescence channel, it was possible to have clear chromatograms on enalapril, as demonstrated in Fig. S2 - UV and FLD.

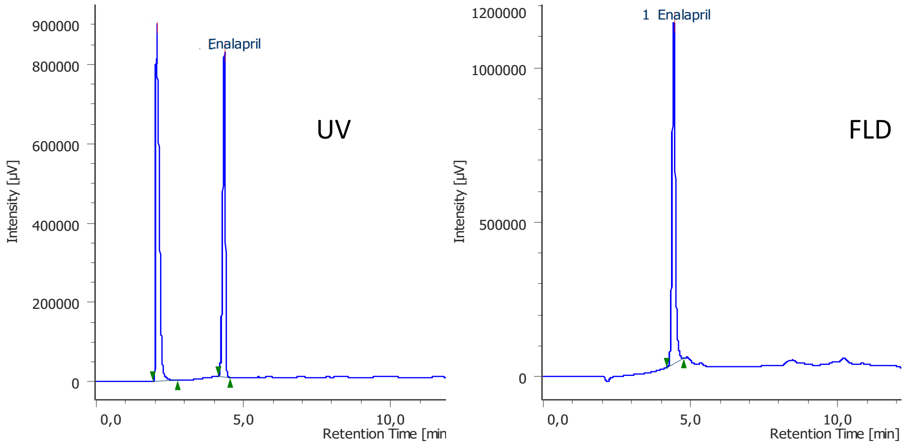

Figure S2. *Chromatogram from an enalapril standard solution detected under UV and Fluorescence detection*

Since the formulations were prepared in a commercial vehicle and complemented by the addition of other compounds, the HPLC gradient programs were adjusted to grant no overlaying of any formula components with those from the drugs.

In the case of Atenolol formulation, all the remaining formula components eluted later than the drug, with complete base separation from the first interference (Fig. S3). Again, fluorescence detection enables a clear confirmation.

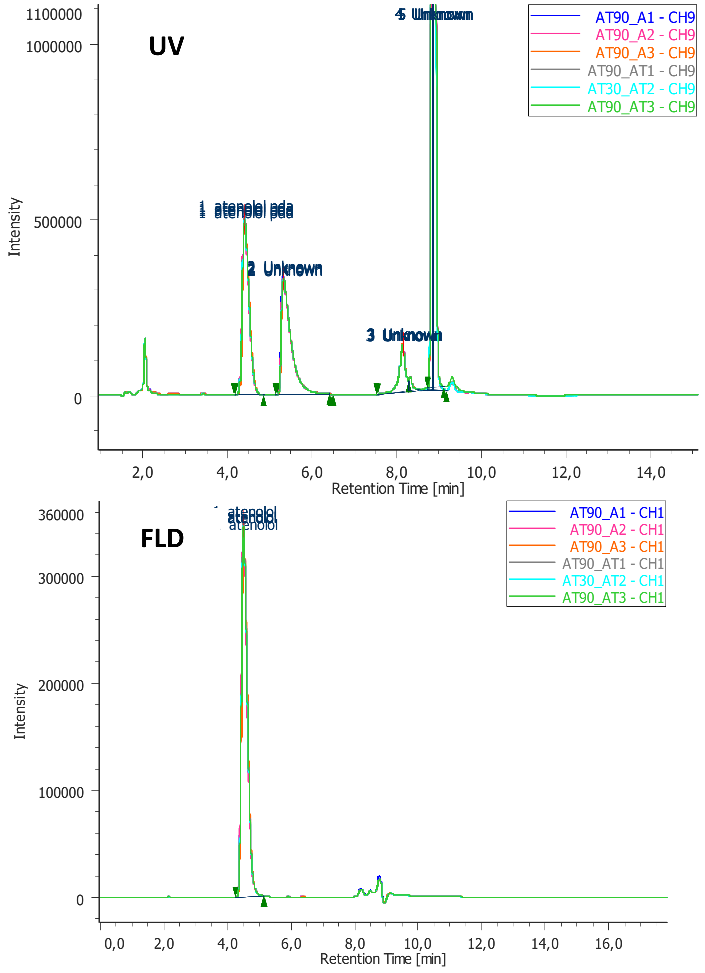

Figure S3. *Chromatograms from six atenolol samples at T90: atenolol eluting at 4.4 min, completely separated from the other matrix components under UV and FLD detections*

For Enalapril Maleate, a clear baseline separation was also achieved from the formulation components, with enalapril eluting at 4.4 min (Fig. S4). In this case, fluorescence readings were also possible, but the chromatographic baseline was not as clear as with atenolol, due to the lower signal response.

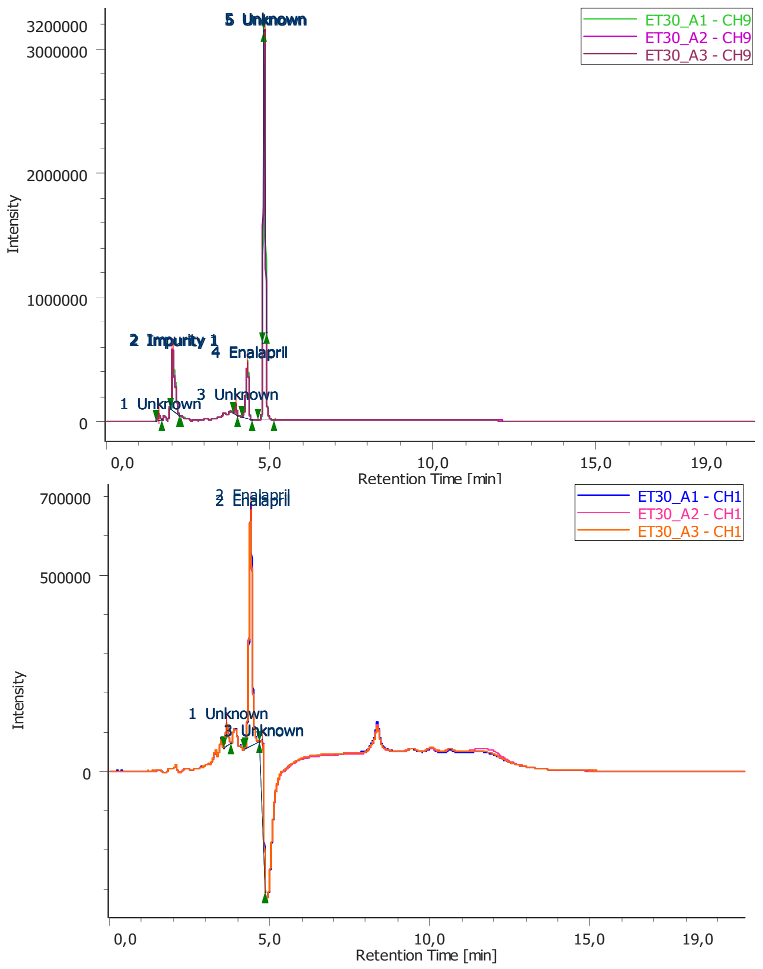

Figure S4. *Chromatograms from a triplicate set of enalapril samples analyzed at day 30, with the UV reading above and the FLD below*

To grant separation from potential interfering peaks resulting from drugs degradation, we have further submitted the drug standards to thermal, acidic and basic stress and photo-degradation. Figure S5 represents overlaid chromatograms (left – atenolol; right enalapril) from the original drug solutions (dark blue) and those resulting from the above stress tests. None of the degradation products co-eluted with the drug standard, under both UV (bottom) and FLD detections (top).

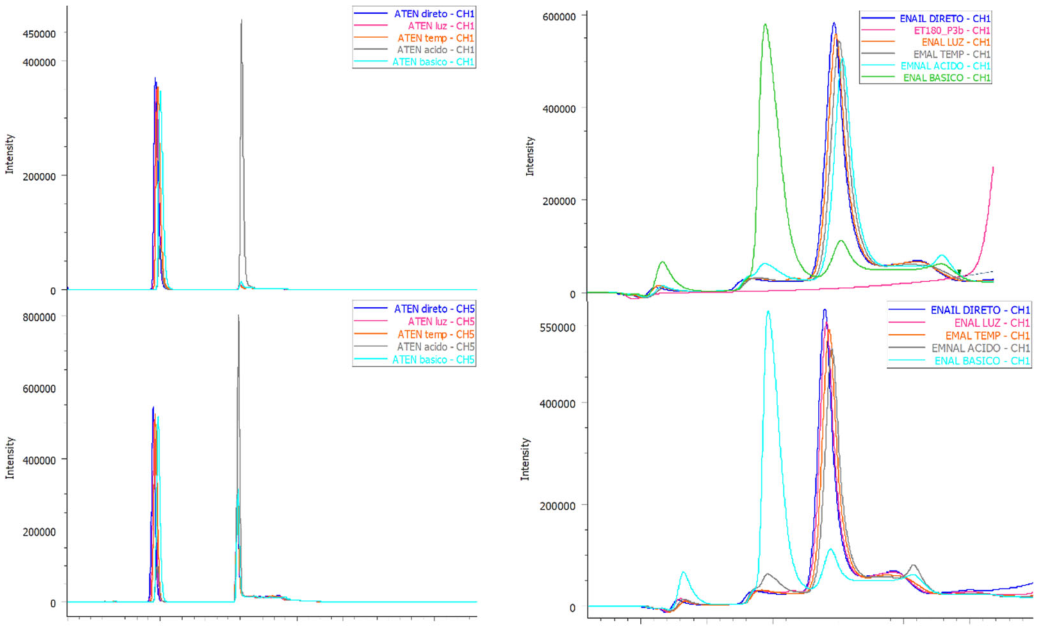

Figure S5. *Overlay chromatograms from the degradation studies, with the UV reading above and the FLD below*

3.2. Stability study of the oral suspensions

3.2.1. Organoleptic characteristics

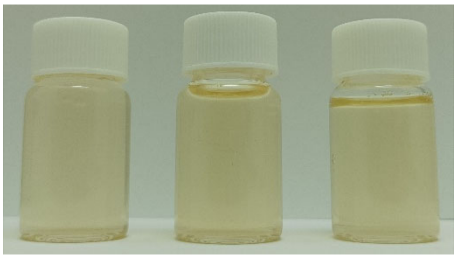

Figure S6. *Appearance of SuspendIt® (left) and atenolol (middle) and enalapril maleate (right) formulations after 180 days storage at 25 °C.*

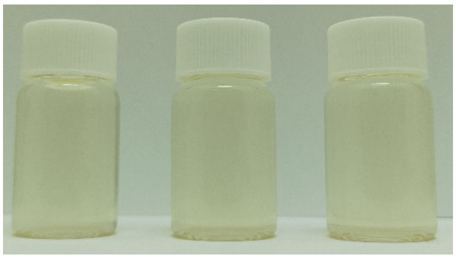

Figure S7. *Appearance of SuspendIt® (left) and atenolol (middle) and enalapril maleate (right) formulations after 180 days storage at 5 °C.*

3.2.3. Rheological properties

3.2.3.1. Flow behavior

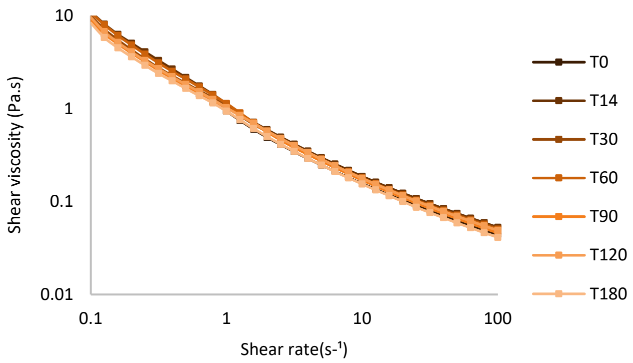

Figure S8. *Flow curve of atenolol oral formulation after storage at 5 °C*

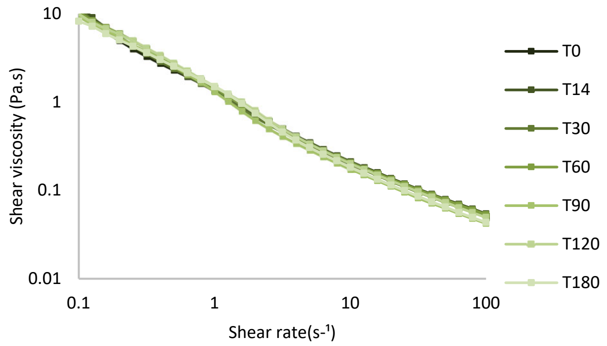

Figure S9. *Flow curve of enalapril maleate oral formulation after storage at 25 °C*

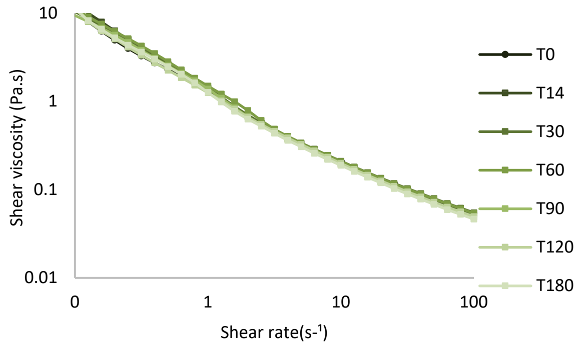

Figure S10. *Flow curve of enalapril maleate oral formulation after storage at 5 °C*

3.2.3.2. Thixotropy

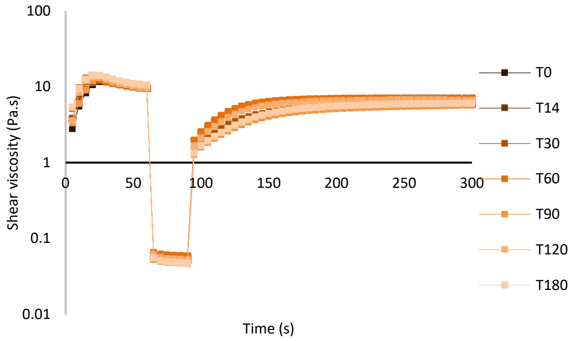

Figure S11. *Thixotropic behavior of atenolol oral formulation after storage at 5 °C*

Table S3. *Recovery period (s) (50%) of atenolol oral formulation (mean ± standard deviation, n=3)*

	**5°C**	**25°C**
**Day 0**	140.00 ± 13.64	
**Day 14**	142.22 ± 9.76	133.89 ± 8.55
**Day 30**	137.22 ± 0.96	142.78 ± 13.57
**Day 60**	136.11 ± 6.74	131.11 ± 9.62
**Day 90**	173.89 ± 49.17	173.89 ± 30.01
**Day 120**	165.55 ± 13.47	162.22 ± 25.07

**Day 180**	180.11 ± 15.01	168.89 ± 20.84

Table S4. *Recovery rate (%) of atenolol oral formulation (mean ± standard deviation, n=3)*

	**5°C**	**25°C**
**Day 0**	69.12 ± 2.34	
**Day 14**	65.57 ± 2.67	69.33 ± 3.54
**Day 30**	66.62 ± 2.86	66.28 ± 2.05
**Day 60**	69.07 ± 3.05	70.10 ± 5.33
**Day 90**	62.90 ± 5.77	61.56 ± 4.85
**Day 120**	60.81 ± 1.77	60.92 ± 5.53
**Day 180**	56.54 ± 3.42	60.47 ± 4.96

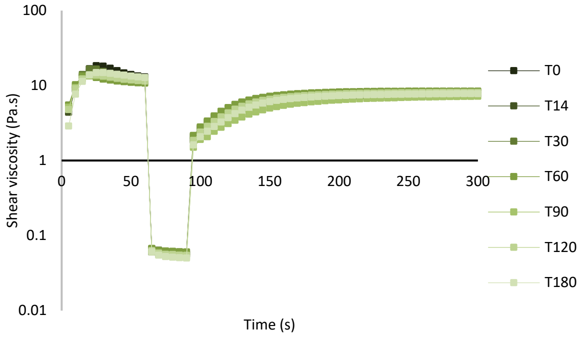

Figure S12. *Thixotropic behavior of enalapril maleate oral formulation after storage at 25 °C*

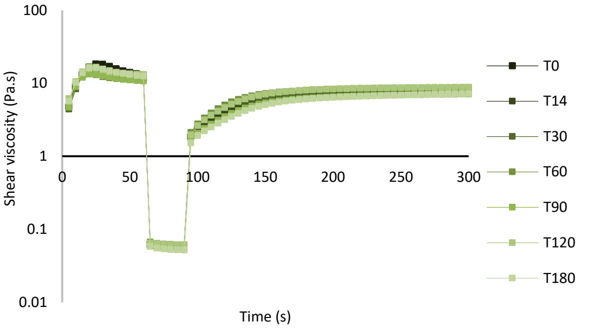

Figure S13. *Thixotropic behavior of enalapril maleate oral formulation after storage at 5 °C*

Table S5. *Recovery period (s) (50%) of enalapril maleate oral formulation (mean ± standard deviation, n=3)*

	**5°C**	**25°C**
**Day 0**	150.56 ± 17.10	
**Day 14**	128.89 ± 6.94	120.56 ± 4.19
**Day 30**	120.56 ± 11.34	141.67 ± 28.87
**Day 60**	133.33 ± 8.66	126.67 ± 2.89
**Day 90**	163.89 ± 10.18	164.45 ± 26.58
**Day 120**	153.89 ± 13.47	143.89 ± 15.49
**Day 180**	165.56 ± 30.01	156.67 ± 10.14

Table S6. *Recovery rate (%) of enalapril maleate oral formulation (mean ± standard deviation, n=3)*

	**5°C**	**25°C**
**Day 0**	64.10 ± 6.99	
**Day 14**	71.19 ± 1.18	70.08 ± 7.47
**Day 30**	77.84 ± 5.74	69.59 ± 4.63
**Day 60**	72.55 ± 5.92	70.04 ± 1.45
**Day 90**	61.80 ± 1.68	62.53 ± 3.99
**Day 120**	63.17 ± 2.57	67.35 ± 9.42
**Day 180**	61.51 ± 3.03	60.71 ± 1.43

2.2.3.3. Amplitude Sweep – Linear Viscoelastic Region (LVER)

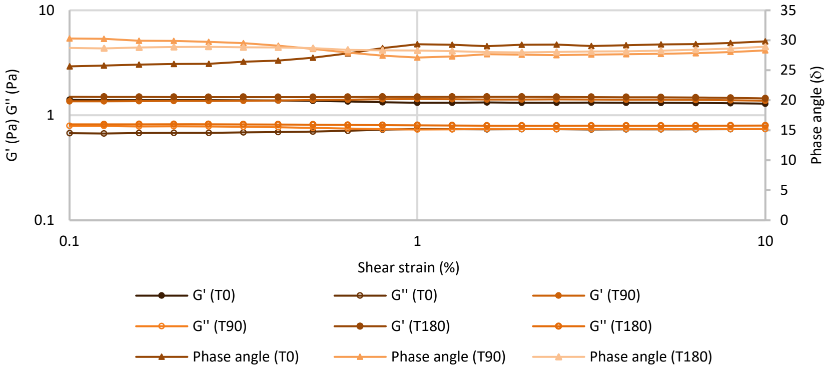

Figure S14. *Amplitude sweep for atenolol oral formulation after 180 days storage at 5 °C*

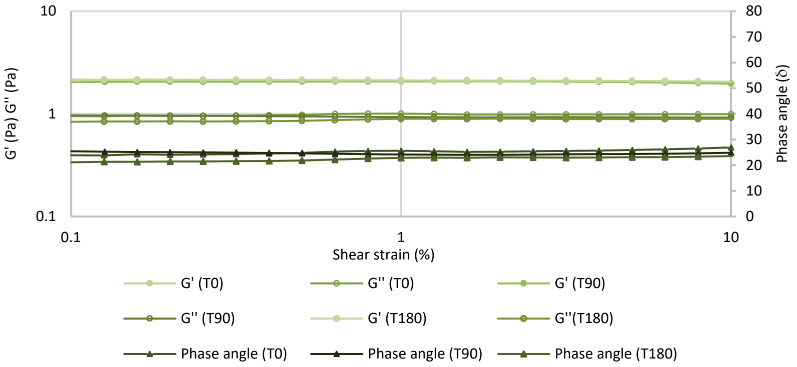

Figure S15. *Amplitude sweep for enalapril maleate oral formulation after 180 days storage at 25 °C*

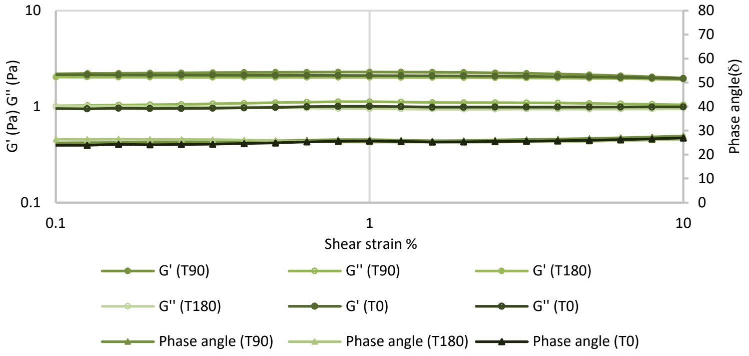

Figure S16. *Amplitude sweep for enalapril maleate oral formulation after 180 days storage at 5 °C*

2.2.3.4. Frequency Sweep (Mechanical Spectrum)

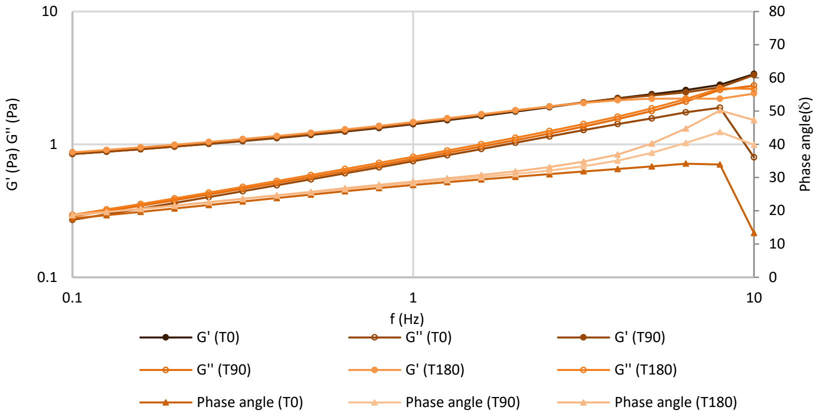

Figure S17. *Mechanical spectrum of atenolol oral formulation after 180 days storage at 5 °C*

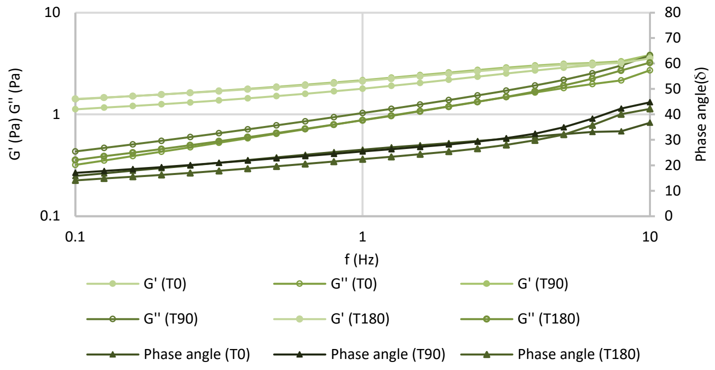

Figure S18. *Mechanical spectrum of enalapril maleate oral formulation after 180 days storage at 25 °C*

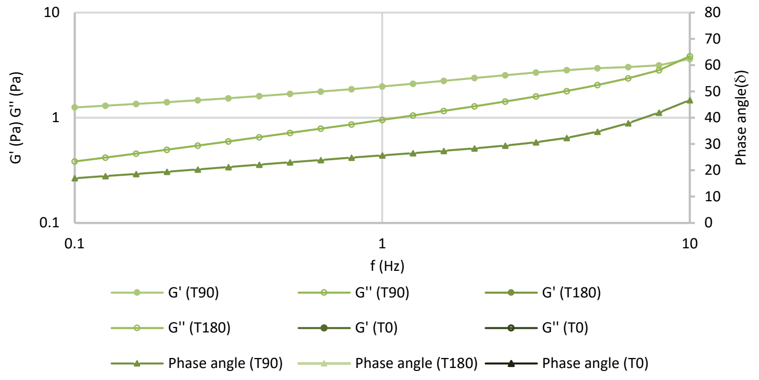

Figure S19. *Mechanical spectrum of enalapril maleate oral formulation after 180 days storage at 5 °C*

3.2.4. Preservative Effectiveness

Table S7. *United States Pharmacopeia Chapter <51> ANTIMICROBIAL EFFECTIVENESS TEST of enalapril maleate oral formulation*

Challenge Microorganism	Day	5°C	25°C
Escherichia coli		Pass*	
Pseudomonas aeruginosa		Pass**	
Staphylococcus aureus	0	Pass*	
Candida albicans		Pass*	
Aspergillus brasiliensis		Pass*	
Escherichia coli		Pass*	Pass*
Pseudomonas aeruginosa		Pass*	Pass*
Staphylococcus aureus	30	Pass*	Pass*
Candida albicans		Pass*	Pass*
Aspergillus brasiliensis		Pass*	Pass*
Escherichia coli		Pass*	Pass*
Pseudomonas aeruginosa		Pass*	Pass*
Staphylococcus aureus	90	Pass*	Pass*
Candida albicans		Pass*	Pass*
Aspergillus brasiliensis		Pass*	Pass*
Escherichia coli		Pass*	Pass*
Pseudomonas aeruginosa		Pass*	Pass*
Staphylococcus aureus	180	Pass*	Pass*
Candida albicans		Pass*	Pass*
Aspergillus brasiliensis		Pass*	Pass*

*>1 log reduction after 14 days
**1 log reduction after 14 days

3.2.5. Active substance assay

Table S8. *Quantification of atenolol by HPLC-UV after storage at refrigerated temperature (n=3, 5 °C)*

Day	0	14	30	60	90	120	180
Mean (mg/mL)	1.99	1.90	1.91	1.90	1.87	1.92	1.84
SD	0.01	0.03	0.01	0.02	0.03	0.01	0.02
%	**99**	**95**	**95**	**95**	**94**	**96**	**92**

Table S9. *Quantification of enalapril by HPLC-UV after storage at refrigerated temperature (n=3, 5 °C)*

Day	0	14	30	60	90	120	180
Mean (mg/mL)	0.50	0.49	0.48	0.48	0.47	0.48	0.48
SD	0.01	0.00	0.01	0.00	0.00	0.01	0.00
%	**101**	**97**	**96**	**95**	**94**	**95**	**95**
